# Injectable Conductive Hydrogels as Multifunctional Bioactive Soft Interfaces for Skeletal Muscle Regeneration

**DOI:** 10.3390/gels12100928

**Published:** 2026-10-15

**Authors:** Xinping Wang, Jingyu Li, Ying Liao, Xiaomin Sun, Zuquan Hu, Lijing Teng

**Affiliations:** 1 Engineering Research Center for Bio-Perception Materials of Guizhou Province, School of Biology and Engineering (School of Modern Industry for Health and Medicine), Guizhou Medical University, Gui’an New District, Guiyang 561113, Chinasunxiaomin@gmc.edu.cn (X.S.); 2 Guizhou Key Laboratory of Microbio and Infectious Disease Prevention & Control, Guizhou Medical University, Gui’an New District, Guiyang 561113, China; 3 State Key Laboratory of Discovery and Utilization of Functional Components in Traditional Chinese Medicine, Guizhou Medical University, Gui’an New District, Guiyang 561113, China; 4 Key Laboratory of Biology and Medical Engineering, Immune Cells and Antibody Engineering Research Center in University of Guizhou Province, Guizhou Medical University, Gui’an New District, Guiyang 561113, China

**Keywords:** injectable conductive hydrogels, soft bioelectronics interfaces, regenerative microenvironment, skeletal muscle regeneration

## Abstract

The skeletal muscle is an essential organ in exercise and energy metabolism and it significantly contributes to maintaining body posture, facilitating movement, and supporting overall health. The skeletal muscle has an intrinsic capacity for self-repair and functional restoration following minor injury. However, this reparative ability is limited, and in cases of volumetric muscle loss (VML) caused by traumatic injury or tumor resection, substantial muscle loss and functional impairments may occur. Injectable conductive hydrogels (ICHs) have received increasing attention in the repair and regeneration of VML due to their controlled conductivity and minimally invasive application. In ICHs, conductive mediums are introduced into a hydrogel to improve the electrical integration between hydrogel and host tissue, promoting skeletal muscle repair. Furthermore, shear-thinning ICHs that undergo in situ gelation can reduce the injury size and inflammation, thus approaching the clinical transformation target of conductive biomaterials. Notably, ICHs can also serve as carriers of bioactive molecules or cells to improve the cellular microenvironment in the injured region and induce skeletal muscle regeneration. This review focuses on exploring different conductive mediums integrated into ICHs, including their formation principles, key performance metrics, immunoregulatory activity, and potential applications in skeletal muscle regeneration. Additionally, this review emphasizes the future development and optimization of ICHs to enhance skeletal muscle repair and regeneration.

## 1. Introduction

Skeletal muscle is essential for body movement, heat production, organ protection, and energy balance. Slightly damaged skeletal muscle can spontaneously regenerate, an intrinsic ability mediated by interactions between satellite cells and microenvironment. However, when severely damaged beyond its intrinsic regenerative capacity, degenerative remodeling and functional decline may occur [1]. Volumetric muscle loss (VML), caused by tumor resection and accidents, can cause extensive necrosis of the surviving functional muscle tissue and fibrotic tissue replacement, resulting in persistent muscle weakness and motor dysfunction [2]. Currently, the benchmark treatment for VML includes autologous transplantation, in which viable, vascularized muscle tissue with a neural supply is surgically transplanted to the defect site. However, autologous transplantation may cause complications at the donor site, postoperative infection, scar tissue formation, and poor regeneration, with additional technical challenges in patients with existing complications [3]. These clinical limitations highlight the urgent need for innovative treatment platforms that can effectively promote new muscle regeneration.

Conductive hydrogels (CHs), which confer biocompatible hydrogels with electrical conductivity, have been developed to facilitate greater interactions between hydrogels and biological systems. CHs can modulate cellular behavior through bioelectrical signal conduction to facilitate tissue regeneration and restore electrophysiological functions [4]. Recent developments have shown that CHs comprising integrated conductive polymers, metal nanoparticles [5], or carbon-based nanomaterials [6] effectively stimulate proliferation, alignment, and differentiation, which are basic processes in the repair and regeneration of skeletal muscle. Minimally invasive injectable conductive hydrogels (ICHs) administered via a syringe or catheter can effectively reduce the injury and inflammation caused by surgery with bulk CHs. ICHs usually rely on a mild cross-linking process based on physical and chemical mechanisms that typically include reversible physical interactions [7] and chemical reactions [7]. ICHs have been developed that integrate both electronic conductivity and minimally invasive techniques for the clinical translation of skeletal muscle regeneration [8]. Moreover, ICHs can also act as bioactive platforms by incorporating cells [9], growth factors [10], exosomes [11], antioxidants [12], or immunomodulatory agents [13] to regulate the regenerative microenvironment [14]. Their conductive properties can also be synergistically coupled with exogenous stimuli, particularly electrical stimulation, to promote myogenic differentiation, angiogenesis, reinnervation, and functional skeletal muscle regeneration [15].

**Figure 1:**
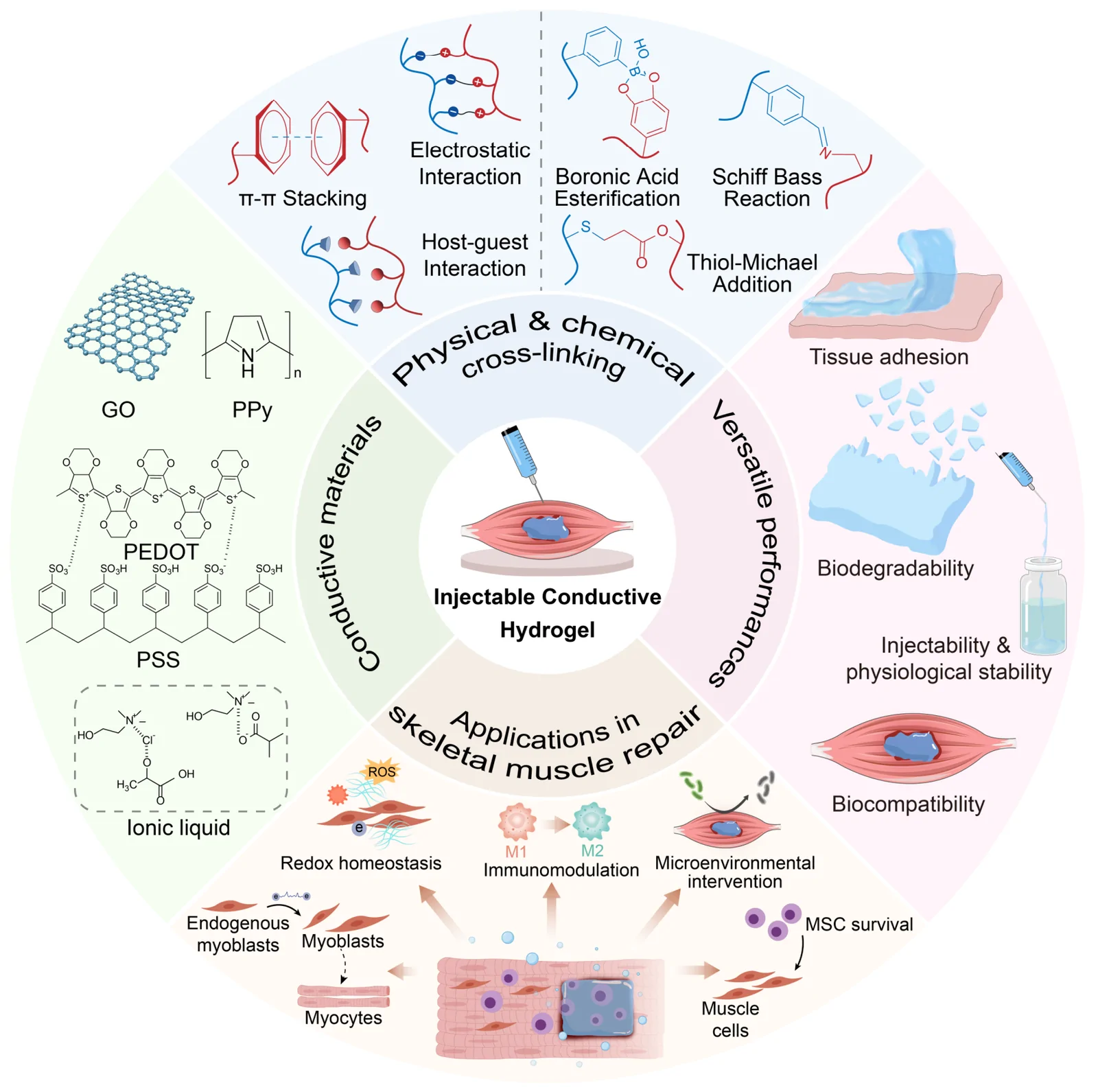
Schematic summary of injectable conductive hydrogels as multifunctional bioactive soft interfaces for skeletal muscle regeneration.

As shown in Figure 1, this review concentrates on engineering ICHs to repair and regenerate skeletal muscle based on the interdisciplinary integration of chemistry, materials science, biology, and medicine. We first highlight the diverse conductive materials used to confer ICHs with electroactivity and biocompatibility, including ions, metals, carbon nanomaterials, and conductive polymers. The construction of ICHs is reviewed from the perspectives of physical and chemical cross-linking, with the structure–property relationships within hydrogel networks highlighted. Furthermore, we discuss the key functional properties of ICHs required for repairing dynamic skeletal muscle defects, including injectability, self-healing, tissue adhesion, physiological stability, biodegradability, and biocompatibility. In addition, we further discuss how bioactive ICHs regulate redox homeostasis, immune responses, bioelectrical signaling, and infection-associated inflammatory microenvironments during skeletal muscle regeneration. Finally, the challenges in the clinical translation of ICHs in skeletal muscle repair and regeneration and future directions are discussed. Distinct from recent reviews that primarily examine electroactive biomaterials [16], electrical stimulation [17], or hydrogel scaffolds from the perspective of a single conductive-material class [18], functional property [19], or regenerative mechanism [20], this review integrates conducting-medium selection, injectable network construction, key interfacial functions, bioactive regulation, and therapeutic applications within a unified “conducting medium-design strategy-function-regeneration” framework. More specifically, we prioritize ICH systems supported by direct in vivo evidence from skeletal muscle-defect and VML models and evaluate them in relation to muscle-specific outcomes, including myogenesis, vascularization, reinnervation, neuromuscular integration, and functional recovery. We also organize the therapeutic roles of ICHs along a hierarchy from local delivery matrices to microenvironment-regulating and bioelectronic interfaces, thereby providing an evidence- and function-oriented perspective beyond material classification alone. This framework clarifies the relationships among material composition, network architecture, multifunctional soft-interface properties, and regenerative outcomes, thereby providing a systematic basis for the rational design and clinical translation of ICHs for skeletal muscle regeneration.

## 2. Conductive Materials for Developing Injectable Conductive Hydrogels

**Figure 2:**
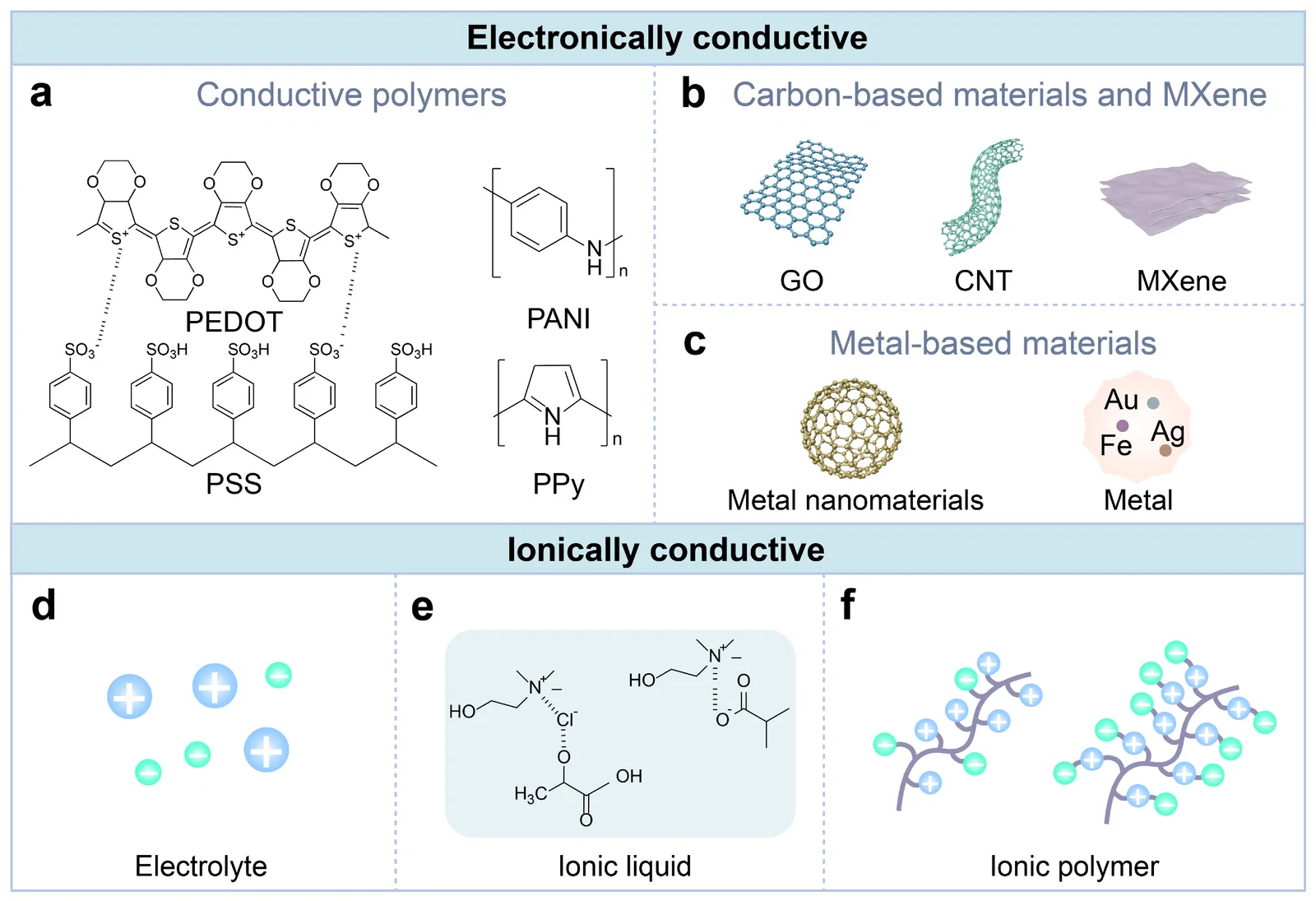
Classification and charge-transport mechanisms of conductive materials for developing ICHs. Electronically conductive components comprise (**a**) conjugated polymers, represented by poly(3,4-ethylenedioxythiophene):poly(styrenesulfonate) (PEDOT:PSS), polypyrrole (PPy), and polyaniline (PANI) in which doping-induced charge carriers move along π-conjugated backbones; (**b**) carbon-based materials and Two-dimensional transition metal carbides and/or nitrides (MXene), represented by graphene oxide (GO), carbon nanotube (CNT), and MXene nanosheets, which conduct through interconnected contact, tunneling, or percolation pathways; and (**c**) metal-based materials, in which free electrons move through continuous or interconnected metallic phases. Ionically conductive components comprise (**d**) free ions/electrolytes migrating through water-rich channels, (**e**) ionic liquids containing mobile organic and inorganic ions, and (**f**) ionic polymers bearing fixed charges and mobile counterions. Electronic and ionic components may be combined to construct mixed ionic–electronic conductive networks that improve tissue–material charge coupling.

Skeletal muscle is a quintessential example of electroactive tissue, with excitation–contraction coupling dependent upon dynamic membrane potential changes, ion channel activation, action potential propagation, and electrochemical signal transmission facilitated by the neuromuscular junction [21]. In cases of severe skeletal muscle injuries, particularly VML, treatment with an injectable hydrogel by itself is typically inadequate for restoring myofiber alignment, myotube maturation, reinnervation, and contractile function [22]. By incorporating conductive elements into injectable hydrogels, electrical integration between the material and host muscle tissue can be enhanced. This integration facilitates the regulation of myoblast proliferation, myogenic differentiation, myotube fusion, angiogenesis, and neurogenesis through electrical signal transmission, while elevating skeletal muscle repair from tissue-filling restoration to comprehensive structural and functional regeneration [15]. Based on the nature of dominant charge carriers, conductive elements integrated into ICHs can be systematically categorized into three primary types: electronically conductive [23], ionically conductive [24], and mixed ionic–electronic conductive systems [25]. Electronically conductive materials facilitate charge transport predominantly through π-conjugated structures [26], carbon nanonetworks [27], continuous metallic phases [15], and two-dimensional conductive nanosheets [28]. Ionically conductive materials mainly depend on the migration of free ions, ionic liquids, or counterions associated with fixed-charge polymers within a hydrogel matrix [24]. Mixed electronic and ionic conductive materials combine both electron transport and ion exchange mechanisms, thereby more accurately recapitulating the charge-transfer processes that occur at interfaces between native tissue and electronic devices [7]. Representative electronically and ionically conductive material classes used to construct ICHs are summarized in Figure 2.

### 2.1. Electronic Conduction

Electronically ICHs enable electrical conduction via continuous or semi-continuous electron transport pathways. The underlying conductive mechanisms include π-electron delocalization along conductive polymer backbones [29], contact or tunneling between carbon nanomaterials or MXene nanosheets [30], and percolative conduction through networks formed by metallic nanoparticles or liquid metals [31]. In comparison to ionically conductive systems, electronically conductive materials typically have superior electrical conductivity and lower interfacial impedance, rendering them particularly useful for electrical stimulation and implantable electroactive scaffolds [24]. However, electronically conductive components are frequently challenged by poor dispersibility, mechanical incompatibility with soft tissue, unclear long-term biosafety, limited biodegradability, and inadequately characterized degradation products [32].

#### 2.1.1. Conductive Polymers

Conductive polymers are among the most extensively used electronically conductive components in ICHs. Notable examples include PEDOT:PSS [33], PANI [34], and PPy [35]. Their electrical conductivity originates from π-electron delocalization on the conjugated polymer chains. Polarons or bi-polarons formed by doping increase the carrier density and enhance charge transport [29]. Compared to inorganic conductive fillers, conductive polymers typically have superior flexibility, processability, and bioelectronic compatibility. Furthermore, in contrast to purely ionically conductive systems, conductive polymers are a more suitable material for constructing low-impedance electrodes and electroactive scaffolds used for electrical stimulation.

Among the various conductive polymers, PEDOT:PSS is a widely studied conductive polymer system. In the PEDOT:PSS system, PEDOT functions as the electronically conductive backbone, with PSS as a polyanionic dopant and dispersing agent that enables PEDOT to form stable aqueous dispersions [33]. With its relatively high conductivity, electrochemical stability, and commercial accessibility, PEDOT:PSS is widely used in applications such as bioelectronics [24], hydrogel electrodes [36], and tissue engineering [23]. However, a significant challenge in ICHs development has not been resolved: concurrently achieving injectability, structural stability, and high conductivity. In response to this challenge, multiple methods have been explored, including freeze-drying with redispersion [37], shear-induced alignment [37], surfactant-induced gelation [38], and supramolecular assembly [39]. These approaches are intended to increase the PEDOT:PSS concentration, promote physical entanglement or π–π stacking, and enable shear-thinning, self-healing, injectable networks [40]. PSS, with its pronounced acidity and water solubility, may adversely affect hydrogel biocompatibility and stability in aqueous environments, and its migration or leaching can progressively compromise the integrity of the conductive network. Although modifying the PEDOT:PSS content or inducing phase separation may enhance conductivity, these modifications frequently suffer from poor elasticity and injectability. To address these challenges, Montazerian et al. substituted PSS with sulfonated alginate (AlgS) to develop water-dispersible and degradable PEDOT:AlgS. This modification enhanced PEDOT dispersion and increased the conductivity [37]. PPy has attracted considerable attention due to its good biocompatibility, simple preparation, and stable electrical performance under physiological conditions. It is frequently combined with collagen, gelatin, gelatin methacryloyl (GelMA), or similar materials through in situ polymerization [41], grafting [42], or metal coordination [43]. PANI and its oligomers, including aniline trimers, tetramers, and pentamers, exhibit reversible redox activity, high antioxidant capacity, and antibacterial potential [23]. However, PANI-based systems also typically have low conductivity, and their reliance on acid doping and inherent hydrophobicity may reduce their long-term stability in physiological environments [44]. To mitigate these potential biosafety concerns, synthesis should combine stoichiometric control with repeated washing or dialysis and quantitative analysis of residual monomers, oligomers, oxidants, and dopants. Conductive-phase content should be limited to the minimum required for target conductivity, and dopants should be selected through formulation-specific cytocompatibility screening [45]. Material-level strategies include linking conductive aniline oligomers to degradable segments or grafting PANI onto degradable biopolymers, as well as preparing purified, uniformly dispersed PPy nanoparticles that are immobilized within a hydrophilic hydrogel network [45,46]. For example, a chitosan-grafted PANI/oxidized-dextran/laponite hydrogel showed more than 45% mass loss after 28 days and greater than 97% cell viability, whereas purified PPy nanoparticles dispersed in gelatin/hyaluronic acid scaffolds exhibited homogeneous distribution and greater than 84% cell viability [35,46]. Nevertheless, long-term implantation studies should quantify conductive-fragment release, biodistribution, macrophage responses, and fibrosis under physiologically relevant electrical and mechanical stimulation.

#### 2.1.2. Carbon-Based Materials and MXenes

The main carbon-based materials are graphene [47], GO [48], reduced graphene oxide (rGO) [49], CNTs [50], carbon nanofibers [51], and associated nanostructures [52]. The electrical conductivity of these materials are primarily ascribed to π-electron delocalization within sp2 carbon networks and the creation of percolative conductive pathways among interconnected nanofillers. In ICHs, GO, rGO, and CNTs are frequently used as conductive fillers and nano-reinforcing agents to improve electrical signal transmission and mechanical stability [50]. Despite these advantages, carbon-based nanomaterials still have challenges. GO, rGO, and CNTs are susceptible to aggregation due to π–π stacking and hydrophobic interactions, which can generate heterogeneous conductive networks, mechanical defects, and localized cytotoxicity [53]. Although the high length-to-diameter ratio of CNTs can produce conductive pathways at low percolation thresholds, their long-term in vivo degradation, potential to elicit inflammatory responses, and associated fibrotic risks require further systematic investigations [53]. These risks can be mitigated by controlling sheet size [54] and oxidation/reduction degree [55], removing residual oxidants, reductants, and metal contaminants [56], restricting the filler content to the minimum level needed to establish a conductive pathway, and coating or functionalizing the nanosheets with hydrophilic polymers or biopolymers before immobilization within the hydrogel network [57]. For example, modifying the surface of rGO with thiol-functionalized Pluronic F127 (F127-SH) improved its aqueous dispersibility and prevented aggregation. The resulting hydrogels exhibited electrical conductivities of approximately 21–22 mS/cm and Young’s moduli of approximately 15–17 kPa, making them suitable for in vivo electromyographic signal recording [57]. In a separate coating comparison, polydopamine produced the strongest short-term improvement in GO/rGO suspension stability, whereas propylene glycol alginate attenuated lactate dehydrogenase (LDH) release from human chondrocytes; the response remained concentration- and coating-dependent [58]. Therefore, coating candidates should be screened in the final injectable composite at the intended dose, with cytocompatibility, inflammatory activation, leachables, degradation, and biodistribution evaluated rather than inferred from the pristine nanofiller.

MXenes are two-dimensional transition metal carbide/nitride materials that combine high electrical conductivity with hydrophilic surface functionality and a large specific surface area [59]. These attributes confer MXenes with surface adsorption [60], photothermal activity [61], antibacterial effects [62], antioxidant functionality [63], and the ability to facilitate electronic transport [64]. For example, Cheng et al. developed Nb_2_C MXene-functionalized ICHs from oxidized pullulan, 3,3′-dithiobis(propionohydrazide) (TPH), and polyethylenimine (PEI)-grafted Nb_2_C MXene@polydopamine (PDA) nanosheets, in which the Nb_2_C MXene provided electronic conductivity, reactive oxygen species (ROS) scavenging, and antibacterial properties, while the PDA coating enhanced the hydrogel stability and tissue adhesion. PEI aids cross-linking and boosts antibacterial effects [28]. However, MXene-based ICHs still have significant challenges related to their stability and biosafety. MXenes, such as Ti_3_C_2_ and Nb_2_C, are prone to oxidation in aqueous and oxygen-rich environments, potentially reducing conductivity and causing structural degradation. The long-term in vivo degradation, metal ion release, biodistribution, and immunological safety of these materials have yet to be comprehensively determined [28]. Furthermore, excessive MXene loading may increase the stiffness of ICHs, reducing their mechanical compatibility with native muscle tissue. Consequently, strategies such as PDA coating [65], antioxidant protection [66], and polymer grafting [37] are needed to improve the stability, bioactivity, and biosafety of MXene-based ICHs.

#### 2.1.3. Metal-Based Materials

Metal-based conductive materials used in ICHs primarily include metallic nanoparticles [67], metallic nanowires [68], and liquid metals [69]. Metallic nanoparticles, such as gold and silver nanoparticles, form conductive pathways through interparticle contacts or synergistic interactions with conductive polymers [70]. With their high aspect ratios, metallic nanowires (such as Ag nanowires) readily form continuous conductive pathways at relatively low filler concentrations [71]. Liquid metals, particularly gallium-based liquid metals, can deform within soft hydrogel matrices while preserving conductive pathways [72,73]. Electrical conduction of metallic nanoparticles, metallic nanowires, and liquid metals occurs when free electrons move rapidly through continuous metallic phases or interconnected contact networks that have high electronic conductivity and low impedance [74]. In addition to their conductive performance, metallic nanomaterials also confer ICHs with antibacterial and photothermal characteristics [23]. This unique set of characteristics positions ICHs with high potential in bioelectronics and the regeneration of electroactive tissue.

Metal-based conductive hydrogels present many advantages in skeletal muscle applications. Ag nanomaterials have broad-spectrum antibacterial activity, which may be beneficial for treating infected muscle defects or facilitating traumatic repair [75]. Au nanomaterials have superior chemical stability and photothermal responsiveness, which can support treatment with externally triggered therapeutics [76]. Liquid metals, with liquid-state deformability, can effectively accommodate mechanical deformation during muscle contraction. However, these materials also pose significant safety and stability challenges. Silver ions and Ag nanoparticles may cause cytotoxicity and provoke dose-dependent inflammatory responses. Certain metallic nanomaterials are non-degradable and may potentially induce chronic immune activation with prolonged retention. Particles may aggregate within the hydrogels, leading to localized increases in stiffness and heterogeneous conductivity [77]. Although liquid metals are mechanically compliant, issues related to their oxide shells, interfacial stability, long-term biodistribution, and degradation behavior require further systematic investigation. Consequently, while metal-based conductive components have the potential to improve conductivity and multifunctionality, it is imperative to optimize their concentration, dispersion, degradability, and long-term biosafety prior to their application in skeletal muscle regeneration [77].

### 2.2. Ionic Types

Electrical conduction in ionically conductive hydrogels occurs when mobile ions migrate within media containing either an aqueous solution or an ionic liquid [78]. This mode of conduction closely mirrors the transmission of electrical signals in native tissue, as the electrical activity in nerve and muscle tissue fundamentally depends on the transmembrane flux of ions, such as Na^+^, K^+^, Ca2^+^, and Cl^−^ [79]. Compared with electronically conductive materials, ionically conductive materials typically have superior biocompatibility, greater flexibility, and more seamless integration with hydrogel networks [78]. However, their conductivity is sensitive to numerous factors, including water content, ion concentration, temperature, pH, swelling, and ion leakage, which collectively affect the long-term stability and low-impedance signal transmission capabilities of ionically conductive materials.

#### 2.2.1. Free Ions (Electrolytes)

Free ion-conductive hydrogels are typically made by incorporating electrolytes, such as NaCl, KCl, CaCl2, LiCl, Fe^3+^, Mg^2+^, and Zn^2+^, into a hydrogel matrix [80]. The free cations and anions migrate through water-rich channels in response to an applied electric field, thereby generating ionic currents. The intrinsically abundant water and interconnected porous structures allow free ions to rapidly diffuse throughout the hydrogel network [81]. Consequently, these systems are easy to prepare, cost-effective, and exhibit a high degree of biocompatibility. Additionally, free ions contribute to network cross-linking and functional regulation [7]. For example, Ca^2+^ initially confers a PEDOT:PSS dispersion with greater injectability and subsequently contributes to the dual-network stability following photo-cross-linking [82]. Fe^3+^-mediated coordination with polyphenols, catechol groups, or carboxyl groups forms dynamic cross-linked networks, thereby imparting self-healing, adhesive, and antioxidant properties to the hydrogels [7]. From a biological perspective, free ion-conductive systems have the potential to replicate the electrolyte microenvironment present in skeletal muscle and help regulate cellular electrical activity [83]. Ca^2+^ is essential for excitation–contraction coupling in muscle tissue, and Na^+^ and K^+^ are fundamental to action potential generation and conduction. Consequently, strategically modulating free ion species and their concentrations could enhance the electrophysiological compatibility between conductive hydrogels and skeletal muscle tissue [83]. However, ion diffusion and leaching from hydrogel matrices may compromise their long-term conductive stability. High salt concentrations may cause an osmotic imbalance, cellular dehydration, or inflammatory responses. Furthermore, ionic conduction is more effective in short-range signal transmission, and its use may be limited in applications requiring low-impedance, long-distance electrical stimulation or electromyographic recording [84]. Consequently, it is preferable to integrate ions with electronically conductive materials or charged polymers to develop composite conductive networks that are stable and efficient [84].

#### 2.2.2. Ionic Liquids

Ionic liquids refer to salts formed by organic cations and organic and inorganic anions, which typically remain liquid near ambient temperature [85]. Compared to traditional aqueous electrolyte solutions, ionic liquids have distinct advantages, such as low volatility, high ionic conductivity, a broad electrochemical window, and excellent thermal stability [86]. Conduction in ionic liquids results from cations and anions migrating through the gel matrix. The formed hydrogels have significant positive attributes, including dehydration resistance and long-term stability [85]. Consequently, ionic liquid hydrogels have strong potential in long-term implantable bioelectronics, flexible sensing platforms, and gel systems resistant to freezing or dehydration. Nevertheless, the use of ionic liquids in injectable hydrogels for skeletal muscle regeneration warrants careful consideration. Certain ionic liquids may cause cytotoxic or membrane-disruptive effects, and their osmolarity, viscosity, and metabolic compatibility can significantly deviate from physiological environments [87]. Future investigations should prioritize the development of bio-derived ionic liquids comprising inherently biocompatible constituents, such as choline, amino acids, lactate, or glucose, to mitigate potential toxicity [86].

#### 2.2.3. Ionic Polymers

Ionic polymers incorporate cationic, anionic, or zwitterionic groups along their molecular chains. Examples of ionic polymers include poly(styrene sulfonate), poly(acrylic acid), alginate, hyaluronic acid, chitosan, heparin, chondroitin sulfate, sulfobetaine, carboxybetaine, and poly(ionic liquids) [88]. The primary conduction mechanism in ionic polymers involves attraction between mobile counterions with fixed charged groups that establish ion transport pathways within a hydrogel network [84]. Ionic polymers reduce the rapid ion loss of free electrolytes while enhancing network cross-linking, antifouling properties, tissue adhesion, and bioactivity [89]. Ionic polymers modulate cell adhesion, inflammatory responses, and growth factor retention. By incorporating these polymers as ionically conductive components or dopants within conductive polymers, electrical conductivity with bioactivity can be integrated. For example, heparin-like structures bind growth factors, hyaluronic acid modulates cell migration and inflammation, and chitosan enhances antibacterial and adhesive properties [90].

### 2.3. Electronic and Ionic Types

Mixed ionic and electronic conductive hydrogels are currently among the most effective material systems for replicating the electrophysiological characteristics of skeletal muscle tissue [91]. In native skeletal muscle, bioelectrical signals are primarily transmitted by ionic fluxes, while external electronic devices use electron transport. Consequently, materials that support both electronic and ionic conduction can increase charge conversion efficiency, reduce impedance coupling, and enable volumetric capacitive charge storage at the interface between the tissue, material, and device. These materials are commonly referred to as organic mixed ionic and electronic conductors [84]. When electronically conductive organic components and ionically conductive phases are both present within a hydrogel network, mixed ionic and electronic conductive gels are formed.

Typical mixed ionic and electronic conductive hydrogels can be broadly categorized into distinct groups. The first group comprises conductive polymers integrated with ionic hydrogel matrices, exemplified by systems that include PPy/GelMA [92], PANI/chitosan [93], heparin-doped PEDOT (PEDOT:HEP) hydrogels [94], and PEDOT:AlgS-containing injectable networks [37]. In these hydrogels, electronic transport is facilitated by conductive polymers, such as PPy, PANI, or PEDOT derivatives, while ion migration is promoted by the aqueous phases, charged polysaccharides, and ionic groups within the hydrogel network. The second group includes carbon-based or MXene electronic networks incorporated into ionic or dynamic polymer networks, such as F127-SH-functionalized rGO (F-rGO)/PEG injectable electrodes and Nb_2_C MXene-functionalized hydrogels [28]. In the F-rGO/PEG injectable electrodes, rGO supports electronic conduction, while the PEG hydrogel matrix and physiological saline environment enable ionic coupling and facilitate electromyographic signal recording. In the Nb_2_C MXene-functionalized hydrogels, electrical conductivity and reactive oxygen species scavenging capabilities are imparted by Nb_2_C MXene [28]. The third group includes multiphase composite systems comprising conductive polymers, metals or liquid metals, and ionic polymers. Notable examples include electroactive injectable hydrogels comprising PEDOT:HEP, liquid metals, polyphenols, and dynamic chitosan/dextran networks [94]. These systems synergistically provide electronic conduction, ion exchange, tissue adhesion, and self-healing capabilities.

Conductive polymer-based hydrogels containing biocompatible polyanionic dopants or ionic polymer matrices also represent mixed ionic–electronic conductive materials [94]. In these systems, electronic transport is enabled by conjugated conductive polymer domains, whereas ion migration and charge compensation are facilitated by polymer chains, mobile counterions, and water-rich channels. Under external electrical stimulation, ions migrate within the hydrogel network and modulate the redox state of conductive polymer domains, generating volumetric capacitance and reducing interfacial impedance [91]. This mixed-conduction mechanism is particularly useful in bioelectronic applications as it converts external electronic signals into localized ionic or electrochemical cues. This mechanism may effectively regulate the myocyte membrane potential, Ca^2+^ signaling, myogenic differentiation, and myotube maturation in skeletal muscle tissue [95]. Electrochemical impedance spectroscopy (EIS) complements bulk conductivity by resolving frequency-dependent resistive and capacitive responses. Nyquist and Bode spectra, including impedance magnitude and phase, should be interpreted using an appropriate equivalent-circuit model to assess percolation resistance and interfacial polarization. For example, PEDOT:AlgS-containing alginate formulations exhibited lower fitted percolation resistance than their PEDOT:PSS comparators [37]. Meaningful comparisons require specification of frequency range, electrode geometry, hydration, and measurement configuration. However, the electrical and mechanical properties of ICHs may change upon exposure to biological environments. Physiological electrolytes and swelling can alter ionic mobility and charge density [96], whereas oxidation of susceptible MXenes may compromise electronic conduction [97]. Furthermore, pH-dependent doping, matrix degradation, and cyclic muscle deformation may destabilize conductive pathways and tissue–material interfaces. These effects depend on material composition and implantation conditions. Therefore, initial conductivity and short-term cytocompatibility should be complemented by longitudinal assessment of electrical, mechanical, and degradation behavior under physiologically relevant conditions, followed by in vivo validation over the intended regeneration period.

## 3. Design Approaches for Injectable Conductive Hydrogels

**Figure 3:**
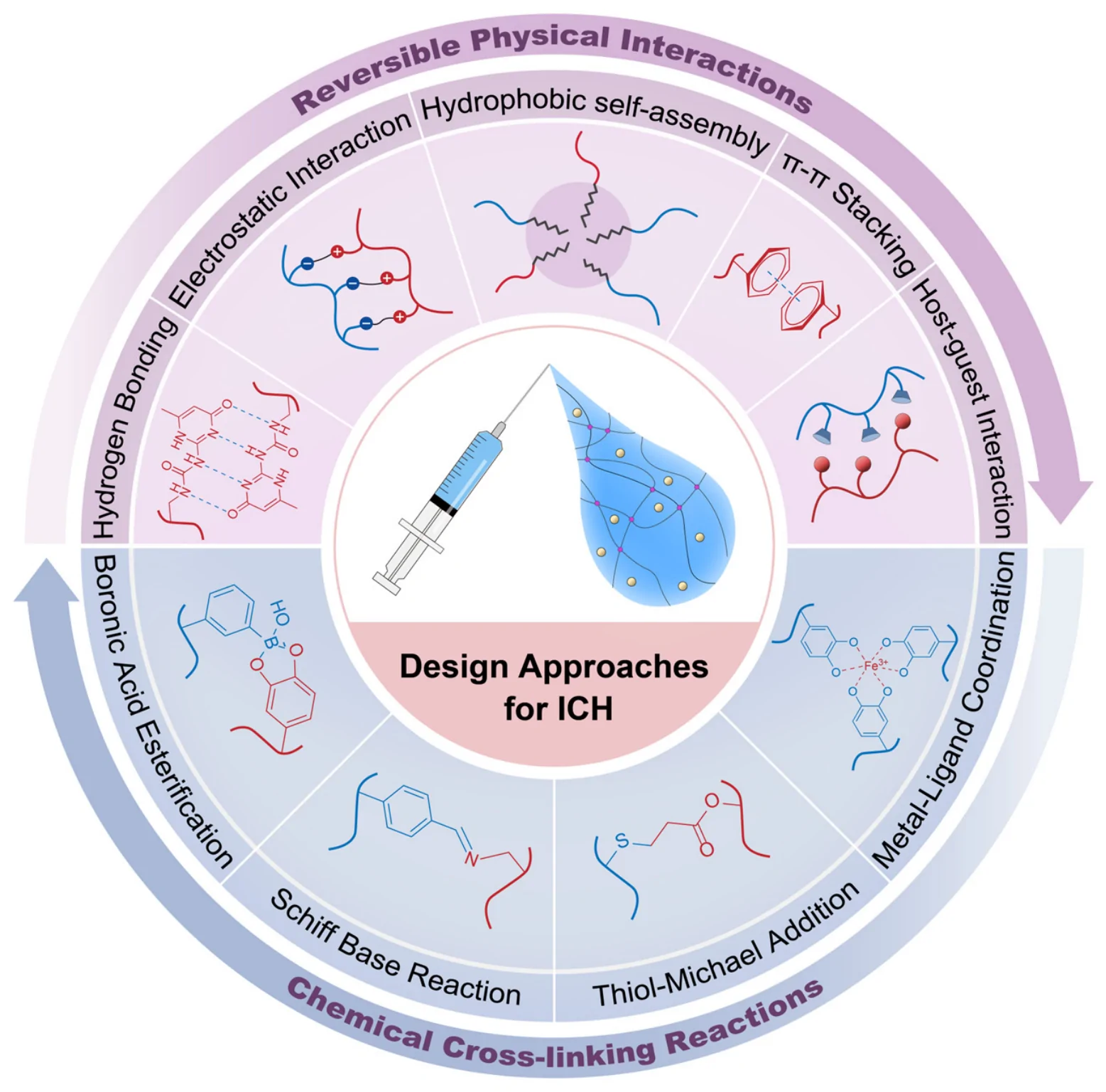
Design strategies for constructing injectable conductive hydrogels. Reversible physical interactions include hydrogen bonding, electrostatic attraction, hydrophobic self-assembly, π–π stacking, and host–guest complexation; these junctions can dissociate under shear and reassociate after injection, thereby supporting shear-thinning and self-healing. Chemical or coordination-based cross-linking routes include boronate ester formation, Schiff base reactions, thiol–Michael addition, and metal–ligand coordination, which provide controllable gelation, mechanical reinforcement, and physiological stability. The integration of reversible physical interactions with covalent or coordination junctions facilitates the formation of physicochemical cross-linked networks, thereby achieving an optimal balance between injectability, structural retention, and the continuity of conductive pathways.

CHs have demonstrated considerable potential in muscle regeneration, as their stiffness, conductivity, and capacity to support bioelectrical communication closely match the functional requirements of electroactive muscle tissue. However, many existing conductive hydrogels have relatively rigid structures that do not easily conform to irregular wound defects and dynamically changing microenvironments, rendering them more suitable for patch-type or surface coating applications. ICHs administered via syringe injection are a promising solution for effectively delivering the hydrogel to the treatment site. Injectable conductive hydrogels can be broadly grouped according to the interactions responsible for network formation as physical, chemical, and physicochemical hybrid systems [18,37]. The major physical and chemical cross-linking mechanisms used to construct ICHs, which may also be integrated into physicochemical hybrid networks, are summarized in Figure 3.

### 3.1. Physically Cross-Linked Injectable Conductive Hydrogels

Physically cross-linked ICHs are primarily constructed by reversible noncovalent interactions, such as hydrogen bonding [98], hydrophobic self-assembly [99], π–π stacking [100], electrostatic interactions [101], and host–guest interactions [102]. These dynamic interactions dissociate under shear stress, leading to a reduction in gel viscosity, which facilitates smooth passage through a needle. Following injection, these reversible interactions reassociate to reconstruct the hydrogel network, thereby endowing the hydrogel with shear-thinning and self-healing properties.

#### 3.1.1. Hydrogen Bonding

Hydrogen bonding arises when a hydrogen atom covalently linked to an electronegative atom interacts with an electron-rich acceptor group within the same molecule or an adjacent molecule [100]. Compared to covalent bonds, individual hydrogen bonds are weak. However, numerous reversible hydrogen bonds can form an energy-dissipating network. This type of network imparts unique properties to hydrogels, including self-healing capabilities, shape-memory behavior, reversible deformation, and shear-thinning characteristics [103]. In ICHs, hydrogen bonds may form between functional groups that provide dual functionality. First, the hydrogen bonds act as cross-linking points between polymer chains, forming a three-dimensional network that allows the conductive hydrogels to flow under shear stress and subsequently recover its structure upon the removal of the shear force [18,37,104]. Second, hydrogen bonds stabilize conductive components [105]. For example, the imine and amine groups present in PANI and PPy can interact with carboxyl, hydroxyl, and amide groups in hydrophilic polymers, stabilizing conductive components within the hydrogel matrix [8,34]. A cellulose nanofiber/poly(vinyl alcohol) network provided abundant hydrogen-bonding sites and dynamic cross-linking domains capable of accommodating PEDOT:PSS, thereby improving the structural stability and mechanical recovery of the conductive hydrogel [106]. The reversible nature of these interactions enables energy dissipation during deformation and facilitates subsequent network reconstruction. However, hydrogels that rely solely on hydrogen bonds for cross-linking may exhibit inadequate mechanical strength or structural fragility, particularly if the hydrogel has a substantial concentration of rigid conducting polymer chains [107]. This limitation is primarily associated with relatively weak and reversible nature of hydrogen bonds, which can dissociate under mechanical stress or in highly hydrated environments, leading to a decrease in effective cross-linking density. Moreover, the incorporation of a high content of rigid conducting polymer chains may create a mechanical mismatch between the stiff conductive domains and the soft hydrophilic matrix, thereby promoting structural heterogeneity, conductive phase aggregation, and localized stress concentration. As a result, the hydrogel network becomes less mechanically robust and more prone to deformation, cracking, or structural failure. To address this limitation, these networks are typically reinforced by multiple hydrogen bonding modes or alternative secondary interactions [108]. Consequently, hydrogen bonding is frequently integrated with other reversible interactions or dynamic covalent bonds to improve mechanical robustness while retaining the network dynamics required for self-healing and injectability [99].

#### 3.1.2. Hydrophobic Self-Assembly

Hydrophobic self-assembly is driven by the propensity of nonpolar groups or hydrophobic polymer segments to reduce their exposure to water, leading to their aggregation into hydrophobic domains. These domains function as reversible physical cross-linking points within aqueous polymer networks [39]. When subjected to shear stress, hydrophobic associations within hydrogel matrix may dissociate or dynamically rearrange, leading to a reduction in network resistance and promoting hydrogel flow. Upon the cessation of the applied stress, the re-association of hydrophobic domains aids in the reconstruction of the network, thereby enhancing the shear-thinning properties, injectability, and self-healing capabilities of the hydrogels [99]. Temperature-responsive phase transition represents another typical manifestation of reversible hydrophobic self-assembly. Lower critical solution temperature (LCST)-type polymers, exemplified by poly(N-isopropylacrylamide) (PNIPAM), remain hydrated and relatively extended below the transition temperature because favorable polymer–water interactions dominate the system [109,110,111]. Upon heating above the LCST, dehydration of the polymer chains weakens polymer–water interactions and enhances polymer–polymer hydrophobic association, resulting in sol-to-gel transition, chain aggregation, and the formation or reinforcement of physical junctions [109,110,111]. This reversible thermally induced association has been widely exploited to regulate phase behavior, network organization, and in situ retention in injectable hydrogel systems [109,112]. Notably, PEG analogue has emerged as a viable alternative to PNIPAM due to its considerably lower monomer toxicity and its highly tunable LCST, which can be adjusted solely through modifications in its structural attributes, such as side-chain length [113]. This obviates the need for copolymerization, a common strategy to modify the LCST in PNIPAM-based materials, which often leads to significantly broadened LCST responses. Additionally, the LCST of PEG analogue exhibits relative stability against variations in the biological environment compared to other LCST polymers [114]. For instance, Zhou et al. described the development of a conductive thermosensitive hydrogel synthesized from PEDOT:PSS/Pluronic F127 through synergistic hydrogen bonding and hydrophobic self-assembly. This hydrogel combines temperature-induced gelation with improved mechanical strength. Such synergy facilitates prolonged mucosal elevation, while the incorporated conductive phase enhances the conduction of electrosurgical currents [115]. Conversely, the gelation process in upper critical solution temperature (UCST)-type systems is governed by the intensification of intermolecular interactions, including hydrogen bonding and electrostatic interactions, alongside the reduced solubility of the polymer in the solvent as the temperature decreases. This mechanism facilitates the aggregation of polymer chains, culminating in the establishment of a stable hydrogel network structure [116]. Gelatin, a well-researched natural polymer, undergoes a thermo-reversible phase transition. When heated above 30 °C, its hydrogen bonds break, unwinding the triple-helix structure into a solution. As it cools, the gelatin partially reforms triple helices through hydrogen bonds and hydrophobic interactions, creating a stable hydrogel network [117,118]. This transition is mainly driven by dynamic intermolecular forces, especially hydrogen bonding [119]. In ICHs, hydrophobic interactions are particularly relevant to conjugated polymers containing intrinsically hydrophobic segments. Appropriate hydrophobic association can enhance interchain contact and facilitate the organization of interconnected conductive domains, while simultaneously providing dynamic junctions for mechanical energy dissipation [38,39,120]. Diverse gelatin-based conductive hydrogel were developed through incorporating different conductive media, the formed conductive hydrogel demonstrates distinct structural configurations in both sol and gel states. At skin temperature, the reformation of the gelatin triple-helix structure results in the formation of a stable hydrogel [121,122,123,124,125]. However, excessively strong hydrophobic association may induce aggregation or pronounced phase separation of conductive components, resulting in structural heterogeneity and potentially compromising aqueous stability and uniform electrical conduction [120]. Therefore, the strength and density of hydrophobic domains should be carefully balanced to maintain both network dynamics and conductive-phase continuity. For example, Zhang et al. developed a room-temperature-formed PEDOT hydrogel using 4-dodecylbenzenesulfonic acid (DBSA). DBSA weakened the electrostatic association between PEDOT and PSS and promoted exposure and association of hydrophobic PEDOT-rich domains, allowing physical network formation through synergistic hydrophobic interactions and π–π stacking. The resulting hydrogel exhibited pronounced shear-thinning, injectability, softness, and self-healing properties [38]. More recently, a related DBSA-mediated assembly strategy was employed to construct an injectable PEDOT:PSS-based conductive hydrogel, in which hydrophobic interactions and π–π stacking contributed to the initial dynamic network before further stabilization under physiological conditions [126]. Collectively, hydrophobic self-assembly provides a useful physical design strategy for simultaneously regulating the dynamic network reconstruction and conductive-domain organization of ICHs.

#### 3.1.3. π–π Stacking

π–π stacking is an important intermolecular noncovalent interaction frequently observed in aromatic or conjugated systems. In addition to π–π stacking between aromatic or conjugated domains, π-mediated forces also include cation–π and anion–π interactions [100]. In ICHs, π–π interactions between conjugated domains can contribute to both physical network formation and conductive-pathway organization. For example, PDA modification of CNTs promoted π–π interactions between aromatic PDA layer and CNT surfaces while simultaneously introducing hydrogen-bonding sites, thereby improving CNT dispersion and interfacial compatibility within hydrophilic polymer networks [127]. Similarly, π–π stacking between PEDOT-rich domains cooperated with hydrophobic association to construct reversible conductive networks in injectable PEDOT:PSS hydrogels [38]. Under shear stress, π–π stacking may be partially disrupted, which decreases the hydrogel viscosity, allowing hydrogel to be injected. Following injection, the aromatic domains reaggregate to restore the network structure. Consequently, π–π stacking serves as an important reversible interaction for regulating hydrogel injectability, conductive-domain organization, and self-healing behavior [38,99]. For example, modifying the surface of CNTs with PDA facilitated π–π interactions and hydrogen bonding that effectively coated the CNTs, enhancing their dispersion within the hydrophilic polymers and minimizing aggregation, which in turn improved the electrical performance and mechanical stability of hydrogel [127]. Kang et al. incorporated lignin in a hydrogel to trigger π–π self-assembly and enabled in situ dual-charge generation to establish highly dipolar and dynamic cross-linking points while increasing the hydrogel mechanical strength and adhesion [128]. Additionally, the in situ assembly of PEDOT:PSS and a zwitterionic polymer formed a stable three-dimensional network through anion–π interactions, π–π stacking, and electrostatic interactions, producing an injectable, self-healing, highly conductive flexible interfacial material [100].

#### 3.1.4. Electrostatic Interactions

Electrostatic interactions, which result from the attraction between oppositely charged groups, are frequently used in systems such as polyelectrolyte hydrogels [129,130,131], alginate-Ca^2+^ complexes [132], PEDOT:PSS composites [37], and charged polysaccharide/protein systems [57,127,128,133]. Electrostatic interactions enable rapid gelation with mild processing conditions and excellent compatibility with aqueous environments. However, fluctuations in pH, salt levels, and physiological ionic environments can reduce structural stability and functional performance of the hydrogels. Electrostatic interactions are therefore often combined with complementary interactions, such as hydrogen bonding [134], hydrophobic interactions [38], and coordination interactions [135]. To reinforce the hydrogel network. Liu et al. used electrostatic interactions and hydrogen bonding to embed PANI nanoparticles within a three-dimensional polymer network. The PANI nanoparticles acted as the conductive phase, and the reinforced interfacial interactions between PANI and hydrogel matrix improved the mechanical properties of system [34]. In a separate investigation, PEDOT:PSS was used as a conductive dopant to cross-link multiple conductive polymers through electrostatic interactions, which markedly enhanced the hydrogel conductivity and biocompatibility [136]. In another study, sodium alginate was introduced into a sulfobetaine methacrylate and acrylamide matrix to generate a distinctive proton transport network, resulting in hydrogels with superior mechanical performance and notable proton conductivity [137].

#### 3.1.5. Host–Guest Interactions

A host–guest interaction is a supramolecular recognition event in which a size-matched and hydrophobic guest molecule is selectively encapsulated within the cavity of a host molecule [102]. This interaction has a complex role in modulating molecular electronic performance [138]. Typical host molecules, including cyclodextrins [139] and cucurbiturils [140], have hydrophilic outer surfaces and hydrophobic cavities that encapsulate hydrophobic guest molecules through noncovalent interactions. In ICHs, hydrophobic electroactive segments, such as aniline oligomers or adamantane-modified conducting polymer complexes, function as guest units. For example, PEDOT:S-Alg-Ad complexes functioned as guest components in a poly(β-cyclodextrin) mediated supramolecular assembly, producing a shear-thinning, self-healing, and biocompatible conductive hydrogel suitable for three-dimensional cell culture [141]. In another representative system, Feng et al. constructed injectable gelatin hydrogels through host–guest complexation between aromatic residues of gelatin and acrylated β-cyclodextrin. The reversible supramolecular interactions provided mechanical resilience, injectability, bio-adhesion, and supported cell infiltration and in situ tissue regeneration [142].

### 3.2. Chemically Cross-Linked Injectable Conductive Hydrogels

Chemically cross-linked ICHs generated under mild conditions using covalent or coordination bonds have structures interconnected by chemical bonds [143,144]. Networks with cross-linking typically have greater stability than those formed purely through physical interactions, rendering them more suitable for long-term applications requiring controllable degradation and high mechanical strength [99]. Optimally, chemical cross-linking should occur in an aqueous environment, proceed under mild conditions, and exhibit low toxicity against cells and tissue. Chemically cross-linked ICHs are commonly fabricated by thiol–Michael addition [145], the Schiff base reaction [146], boronic acid esterification [147], and metal–ligand coordination [148].

#### 3.2.1. Thiol–Michael Addition

Thiol–Michael addition is a nucleophilic conjugate addition reaction between thiol groups and electron-deficient carbon–carbon double bonds [149,150]. With its high efficiency under physiological conditions, the reaction is commonly used to generate mild and controllable cross-linked hydrogel networks. The reaction mechanism fundamentally involves the deprotonation of a thiol by a base or nucleophile under weakly alkaline or near-physiological pH conditions, forming a thiolate anion. The thiolate anion subsequently attacks the electron-deficient double bond of a Michael acceptor, forming a thioether-type addition product [150]. Common Michael acceptors include maleimide [151], vinyl sulfone [152], acrylate [153], and methacrylate groups [154]. While thiol–Michael addition is not inherently conductive, its mild, efficient, and adaptable covalent network-forming capabilities are highly suitable for producing ICHs. Specifically, conductive oligomers or macromolecules can be engineered as multifunctional macromonomers bearing acrylate [155], methacrylate [156], vinyl sulfone [152], or maleimide [157]. The macromonomers then react with thiol-containing biomacromolecules such as hyaluronic acid, polyethylene glycol, or gelatin. C-S covalent bonds are formed that effectively immobilize conductive segments within the hydrogel network, minimizing the aggregation, migration, and leakage of conductive components and creating more uniform and stable conductive pathways [157,158]. For example, Wang et al. developed an injectable conductive hydrogel through in situ Michael addition between a multiarmed tetraaniline–polyethylene glycol diacrylate conductive cross-linker (TA-PEG) and thiolated hyaluronic acid (HA-SH), thereby integrating the electroactive segments directly into the covalent hydrogel network [158]. In a distinct strategy, Yang et al. constructed an injectable covalent network through thiol–maleimide click chemistry between PEG-8SH and PEG-2Mal. Tannic acid was introduced as a molecular regulator to modulate the gelation kinetics, while simultaneously enhancing noncovalent interactions within the MXene/PEDOT:PSS conductive network [157].

#### 3.2.2. Schiff Base Reaction

In Schiff base chemistry, dynamic covalent imine bonds (C = N) are formed through a reaction between aldehyde or ketone groups and primary amines [159]. Unlike permanent covalent bonds, imine linkages can form under mild aqueous conditions and undergo reversible dissociation and reconstruction in response to external stimuli or network disruption. Consequently, Schiff base hydrogels are frequently self-healing, injectable, and pH-responsive [99]. In ICHs, the Schiff base reaction does not inherently confer conductivity; instead, it primarily facilitates the construction of a dynamic covalent network. Conductivity is typically derived from grafted or incorporated electroactive components such as PANI, PPy, PEDOT, MXene, or graphene [23]. A prevalent design approach uses aldehyde-containing polymers, such as oxidized hyaluronic acid [160], oxidized dextran [161], or oxidized alginate [162], which react with polymers containing amino, hydrazide, or aminated conductive segments. Upon mixing, dynamic C = N bonds are formed, and the conductive segments are immobilized within the hydrophilic matrix. This enhances the uniformity of the conductive network and minimizes component leakage [160]. Bi et al. constructed an ICH through Schiff base chemistry using curcumin-loaded artificial melanin nanoparticles (AMNP@Cur), polyaniline-grafted quaternized chitosan (PQCS), and oxidized dextran (OD) as the main components. The resulting PQCS/OD/AMNP@Cur hydrogel exhibited favorable ROS scavenging, conductivity and antibacterial efficacy with the controlled release of curcumin [163].

#### 3.2.3. Boronic Acid Esterification

Boronic acid esterification refers to a dynamic covalent process in which boronic acid or boronate groups interact with cis-diol structures to generate reversible B–O bonds [164]. Typical systems include phenylboronic acid-diol [164], borax-polyvinyl alcohol [165], and boronic acid-modified polysaccharide-PVA [166]. Due to the abundance of hydroxyl or vicinal diol structures in polyvinyl alcohol [167], polysaccharides [168], and catechol-containing molecules [169], these materials provide numerous cross-linking sites for boronic acid esterification [164]. Boronate ester bonds are dynamically reversible and environmentally responsive. This confers hydrogels with injectability and shear-thinning, self-healing, and stimuli-responsive behaviors when subjected to shear or damage, as these bonds can break and reform [164]. In ICHs, boronic acid esterification is crucial for constructing dynamic cross-linked networks. Conductivity is typically imparted to hydrogel by components such as MXene [166], graphene [170], Ag/MXene [171], conducting polymers [172], or ionic conductive elements [173]. Therefore, the boronate ester bonds primarily facilitate dynamic gelation, self-healing, and post-injection network recovery, while conductive fillers or conducting polymers are responsible for transmitting electrical signals [147,166]. For example, reversible boronate ester formation between polyvinyl alcohol and phenylboronic acid-grafted oxidized hyaluronic acid supported self-healing, flexibility, and conformal network reconstruction. MXene@Mg^2+^ nanosheets additionally provided electrical conductivity and NIR-responsive photothermal antibacterial activity, while the incorporated Mg^2+^ contributed to angiogenic and regenerative functions [166].

#### 3.2.4. Metal–Ligand Coordination

Metal–ligand coordination is a dynamic interaction between metal ions and ligands with lone pair electrons. Notable examples include catechol-Fe^3+^ [174], carboxylate-Ca^2+^ [132], imidazole-Zn^2+^ [175], and thiol-Ag^+^ complexes [176]. Coordination bonds formed between metals and ligands typically display greater binding strength than most hydrogen bonds or conventional ionic interactions. These bonds also show dynamic characteristics that support reversible dissociation and reformation. Accordingly, coordination bonds enhance mechanical performance, strengthen adhesive properties, and improve self-healing of hydrogels [177,178]. Owing to these dynamic and reinforcing effects, metal–ligand coordination offers several advantages for the construction of ICHs. First, coordination interactions can function as reversible sacrificial bonds, allowing the hydrogel network to dissipate mechanical energy and recover after deformation. Coordination bonds can exhibit higher binding strength than individual hydrogen bonds and many conventional electrostatic interactions while retaining reversible ligand-exchange behavior [99]. Consequently, appropriately designed coordination junctions can act as reversible sacrificial bonds that dissipate mechanical energy and subsequently reconstruct after deformation. This dynamic feature allows hydrogel network to dissipate mechanical energy, recover after injection, and preserve structural integrity under repeated deformation [174,175,177,178]. In addition, the coordination strength and exchange kinetics can be regulated by the type of metal ion, ligand structure, coordination valence, pH, and metal-to-ligand ratio [179,180]. This tunability provides an effective strategy for modulating hydrogel stiffness, stress relaxation, tissue adhesion, self-healing behavior, and degradation profiles [181]. In ICH systems, metal–ligand coordination can also improve the interfacial compatibility between conductive components and polymer networks, reduce conductive phase aggregation, and help maintain conductive pathway continuity under dynamic deformation [157,176]. By anchoring conductive polymers or nanofillers within the hydrogel matrix, coordination interactions may reduce conductive phase aggregation and migration, thereby contributing to more stable conductive pathways under dynamic physiological conditions [157,179].

Nevertheless, metal–ligand-coordinated ICHs still face several limitations. The stability of coordination bonds is highly dependent on the surrounding physiological environment. Variations in pH, ionic strength, redox state, and the presence of competing ligands, such as phosphate ions, citrate, amino acids, or proteins, may weaken metal–ligand interactions and accelerate network dissociation. Excessive coordination can also increase the cross-linking density and stiffness of hydrogel, which may compromise injectability, stress relaxation, cellular infiltration, and mechanical compatibility with soft skeletal muscle tissue. Moreover, uncontrolled metal ion release may induce cytotoxicity, oxidative imbalance, inflammatory responses, or long-term biosafety concerns [99]. Therefore, the type and concentration of metal ions, ligand density, coordination stability, and metal ion release kinetics should be carefully optimized when designing metal–ligand-coordinated ICHs for skeletal muscle regeneration. Within ICH systems, metal–ligand coordination may cooperate with the conductive phases, including PPy, PANI, PEDOT:PSS, and MXene, reinforcing structural stability and enhancing functional performance. Moreover, catechol-modified conductive polymers and biopolymers can coordinate with Fe^3+^ to rapidly form injectable networks, with favorable tissue adhesion, mechanical strength, and self-healing performance [175,179]. For example, a dual-network hydroxypropyl chitosan (HCS)/2,3,4-trihydroxybenzaldehyde (TBA)/Fe^3+^/pyrrole (Py) hydrogel was constructed in a one-step process. Fe^3+^ served as an initiator for the in situ oxidative polymerization of pyrrole within the hydrogel network, endowing the resulting hydrogel with high conductivity, injectability, self-repair properties, and antibacterial and antioxidant activity [182]. Shin et al. developed injectable conductive granular hydrogels by assembling hyaluronic acid microgels containing gallol moieties. In situ metal reduction generated conductive metal nanoparticles, while gallol–metal coordination at the jammed microgel interfaces contributed to network integration and electrical conduction. The resulting granular hydrogels could be readily extruded through a syringe and were able to electrically bridge separated muscle tissues [148].

### 3.3. Physicochemical Cross-Linked Injectable Conductive Hydrogels

Physicochemical cross-linked ICHs are characterized by reversible physical interactions and chemical cross-linking structures simultaneously incorporated within a single hydrogel network [18,23]. In physically cross-linked systems, network formation is driven by dynamic interactions, such as hydrogen bonding, aromatic π-associated interactions, electrostatic attraction, and supramolecular host–guest recognition [99]. These interactions create strong interfacial adhesion between conductive fillers and hydrophilic polymers, thereby mitigating the aggregation and migration of conductive materials [37]. In contrast, chemical cross-linking involves mechanisms such as Schiff base bonds, thiol–Michael addition, boronate ester bonds, and metal coordination. Chemical cross-linking reinforces the hydrogel framework by linking polymer chains into a stable three-dimensional architecture to maintain the structural integrity of the hydrogel in environments containing bodily fluids, accommodate tissue movement, and facilitate electrical stimulation [157]. Compared to a single cross-linked network, a physicochemical cross-linked network benefits from diverse interactions [108]. Furthermore, physicochemical cross-linking, in addition to generating diverse bond types, produces synergistic structures that integrate the conductive phase, flexible hydrogel network, and tissue interface [183]. For example, ICHs were developed by forming Schiff base linkages and hydrogen bonds between HCS and TBA [182]. In this system, Fe^3+^ coordinated with TBA to introduce reversible metal–ligand interactions, which subsequently triggered the in situ oxidative polymerization of pyrrole inside the hydrogel matrix, generating PPy and giving rise to a double cross-linked conductive network. The resulting hydrogel exhibited injectability, electro-responsive behavior, self-repair, antibacterial and antioxidant activity, and electrical conductivity [182]. Zhang et al. engineered an ICH incorporating PEG-8SH and PEG-2Mal using a mixed gelation process to produce a covalently cross-linked network through thiol–Michael addition. At the same time, the conductive phase and interfacial regulatory structure were generated through the synergistic interaction of MXene, PEDOT:PSS, and tannic acid (TA). The MXene/PEDOT:PSS/TA dispersion then combined with PEG-8SH and PEG-2Mal precursors to generate the ICHs. The resulting ICHs exhibited remarkable injectability and conductivity while providing mechanical and electrical stability [157]. The essence of physicochemical cross-linking lies in the functional synergy of its components: dynamic physical interactions ensure injectability and self-healing, chemical cross-linking provides structural stability, and the conductive phase enables electrical signal transmission. This synergistic design allows the ICH injectability, conductivity, mechanical strength, degradation, cytocompatibility, and tissue adhesion to be simultaneously regulated.

Table 1 compares representative compositions and the advantages and limitations of the cross-linking mechanisms discussed above. Physical junctions favor reversible flow and recovery but may require reinforcement for tissue retention; covalent or coordination junctions provide complementary stabilization, whereas dynamic covalent bonds enable remodeling. Hybrid networks combine these functions, although compositional complexity complicates optimization and attribution of biological effects [99,157].

Degradation requires formulation-specific comparison. OGGP3 exhibited 65.4% weight loss after 12 weeks in PBS at 37 °C [146], whereas ICAA showed no significant mass loss over 4 weeks under comparable incubation conditions [157]. After 25 days of subcutaneous implantation, DECPLMH retained <30% of its MRI-derived implant volume, but >75% of the µCT-detectable material remained, indicating slower liquid-metal clearance [144]. Thus, partial matrix degradation, conductive-filler clearance, and stability over an observation period are distinct endpoints; cross-linking class alone does not define a universal degradation time.

Target tissues and evaluation models also determine the strength of regenerative evidence. Host–guest PEDOT hydrogels supported C2C12 myotube-like formation in vitro [141], whereas Schiff-base HA–PANI hydrogels were evaluated with C2C12 cells and human adipose-derived stem cells and demonstrated repair in a rat tibialis anterior defect model [160]. FPGM additionally provided in vitro and in vivo evidence of myogenic, vascular, and neuromuscular benefits [66]. By comparison, cardiac studies using H9C2 cells and rat cardiac fibroblasts [146], or neural-interface studies using PC12 cells and rat vagus-nerve stimulation [157], provide application-specific design precedents. Consequently, persistence and biological assays should match the intended tissue rather than a universal ranking of ICHs.

Beyond the cross-linking strategies summarized in Table 1, the practical performance of ICHs also depends on gelation kinetics, injectability, yielding behavior, and electrical properties after injection. Table 2 compares these parameters across representative ICH systems and highlights the limited availability of paired pre- and post-injection conductivity measurements.

## 4. Versatility of Injectable Conductive Hydrogels

**Figure 4:**
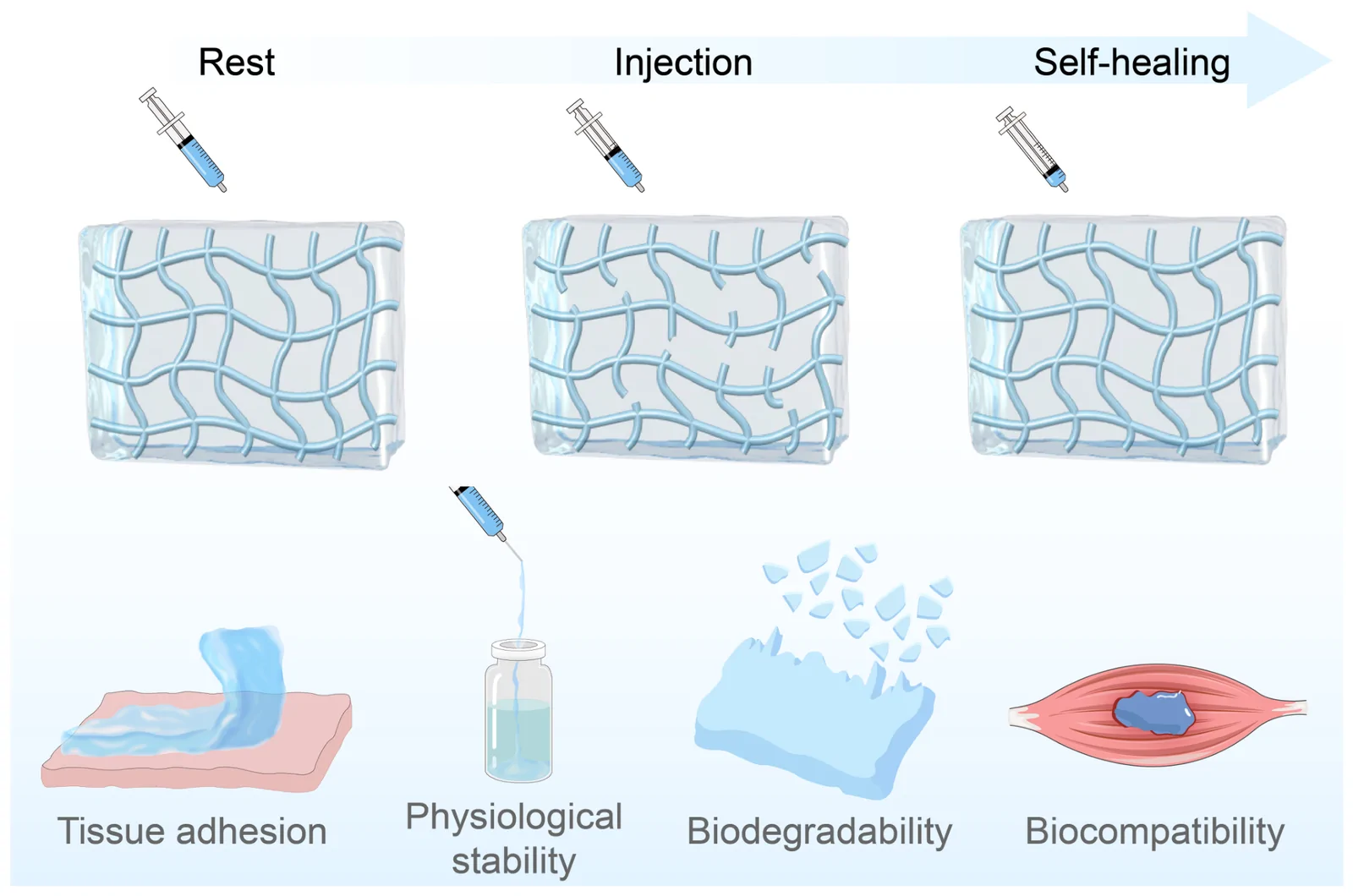
Key functional properties of injectable conductive hydrogels for skeletal muscle repair. At rest, the cross-linked network maintains a stable gel structure; during injection, reversible junctions dissociate and the viscosity decreases, permitting extrusion through a needle; and after shear removal, network reformation restores the gel and its conductive pathways. Tissue adhesion promotes wet interfacial contact and local retention within irregular muscle defects, whereas physiological stability limits excessive swelling, dissolution, and conductivity loss in body fluids. Controlled biodegradation should match the timescale of tissue regeneration, and biocompatibility should support myogenic, vascular, neural, and immune-cell responses without provoking cytotoxicity or chronic inflammation. These properties must be jointly optimized because improving one function may compromise another.

Skeletal muscle injuries, particularly VML, are frequently associated with irregular tissue defects, local inflammation, oxidative stress, inadequate vascularization, impaired neural reinnervation, and persistent mechanical strain due to dynamic muscle contractions [185]. The key advantage of using ICHs to treat skeletal muscle injuries lies in their ability to combine controllable electrical conductivity with minimally invasive delivery, thereby mitigating secondary damage and inflammatory responses that typically occur with surgical implantation. Specifically, electrical conductivity of the ICH matrix provides the required function for use in skeletal muscle repair, while other hydrogel attributes, such as shear-thinning behavior [186], self-healing properties [99], tissue adhesion [187], physiological stability [188], degradability [57], and biocompatibility [189], collectively determine the effectiveness of the ICH for supporting skeletal muscle structural repair and functional restoration under complex in vivo conditions. As summarized in Figure 4, effective ICHs require coordinated shear-thinning and self-healing behavior together with tissue adhesion, physiological stability, biodegradability, and biocompatibility.

### 4.1. Shear Thinning

Shear thinning is an essential characteristic for achieving minimally invasive administration of ICHs. Shear-thinning ICHs exhibit reduced viscosity when subjected to high shear stress, allowing the hydrogel to smoothly extrude through needles, catheters, or printing nozzles. When the shear force is removed, shear-thinning hydrogels revert to their gel-like state, thereby ensuring local retention and spatial filling at the defect site [186]. This attribute is particularly critical in VML treatment, in which the defect geometry is often irregular and challenging to accommodate using a bulk hydrogel. The shear-thinning properties of CHs typically arise from dynamic reversible networks comprising physical interactions and dynamic covalent bonds. These interactions can undergo reversible cleavage and recombination, thereby imparting the hydrogels with injectability and shape recovery characteristics. For example, Nb_2_C MXene-functionalized ICHs were developed by interconnecting oxidized pullulan, TPH, and PEI-grafted Nb_2_C MXene@PDA nanosheets via Schiff-base, acylhydrazone, and disulfide bonds. The prepared ICHs exhibited pronounced shear-thinning behavior, with the viscosity decreasing as the shear rate increased. After injection, the dynamic covalent network reconstructed, enabling the hydrogel to stably conform to the defect site [28].

Injectability is evaluated by a systematic assessment of parameters, such as viscosity, shear stress, shear rate, yield stress, storage modulus, loss modulus, and thixotropic recovery [186]. During the injection process, hydrogels experience deformation due to external shear stress, with flow behavior influenced by changes in viscosity due to varying shear rates. Ideally, ICHs should exhibit the characteristics of non-Newtonian pseudoplastic fluids, evidenced by a substantial decline in viscosity as the shear rate increases. Under high shear conditions, dynamic cross-linking points are temporarily disrupted, facilitating the transition of the gel into a flowable state in which injection resistance is diminished. Post-injection, these dynamic interactions gradually recover, allowing the stable hydrogel network to be reconstructed [99]. Shear-thinning behavior can be directly evaluated from a viscosity–shear rate curve, in which a decreased viscosity at higher shear rates reflects the reversible breakdown of the dynamic network. If the viscosity is excessively low, the material may be easily injected but challenging to retain at the defect site. Conversely, an excessively high viscosity may produce a high injection pressure, needle clogging, and even secondary tissue damage [186]. For example, PEG-peptide/PEDOT:PSS hydrogels establish homogeneous supramolecular networks through synergetic electrostatic interactions, π–π stacking, and anion–π interactions. These hydrogels exhibit a reduced viscosity with an increased shear rate and revert to their gel state upon the removal of shear forces [186]. Yield stress is another crucial parameter for assessing both the injectability and local retention of hydrogels. Yield stress is the minimum stress necessary to initiate irreversible flow in a substance. In VML repair, a yield stress that is too low may result in the loss of ICHs due to tissue fluid dynamics or muscle movements. Conversely, a yield stress that is too high may impede the extrusion of ICHs through narrow needles or catheters [186]. Zhao et al. used a combination of freeze-drying and redispersion to enhance the concentration of PEDOT:PSS nanofibers in an ICH, thereby converting the system from a liquid state to a gel-like material with distinct shear-thinning and shear-yielding properties. During the extrusion process, the use of smaller nozzles caused the PEDOT:PSS nanofibers to align, which enhanced the conductivity of the extruded hydrogel [7]. Accordingly, post-injection conductivity should be measured against matched non-extruded controls after equivalent gelation and hydration, with needle gauge, extrusion rate, and recovery time specified. Rheological recovery alone does not establish restoration of conductive pathways. Therefore, ideal ICHs should have a specific viscosity range and moderate yield stress. This ensures hydrogel integrity under low shear rates while allowing for rapid flow under high shear conditions. Additionally, the ICHs should resist gravitational forces, tissue fluid flushing, and deformation caused by muscle contractions following injection.

### 4.2. Self-Healing

Self-healing is a crucial property that allows ICHs to preserve their structural integrity and conductive continuity within the dynamic environment of skeletal muscle. Skeletal muscles are subjected to continuous contraction, stretching, compression, and shear deformation during routine movements [185]. Consequently, ICHs implanted or injected into muscular defects experience repeated mechanical stresses. If the ICH is irreversibly fractured under cyclic stimuli, the scaffold structure, conductive pathways, and local delivery functions may rapidly deteriorate [99]. Accordingly, self-healing ICHs should recover their macroscopic morphology following damage and restore network connectivity and electrical signal transmission at the microscopic level [7]. Similarly to the shear-thinning behavior of ICHs, the self-healing capability results from reversible interactions, primarily non-covalent interactions and dynamic covalent bonds. These interactions enhance structural recovery through bond exchange and reversible recombination mechanisms [99].

Oscillatory rheological analysis is an important method for evaluating ICH network structure and post-injection recovery behavior. The storage modulus (G′) represents the ability of the material to store elastic energy and sustain a solid-like structure, whereas the loss modulus (G″) represents viscous flow and energy dissipation [186]. Typically, G′ > G″ indicates a gel state, while G″ > G′ suggests a sol state. Frequency sweeps conducted within the linear viscoelastic region can be used to evaluate network stability, while strain sweep measurements reveal the critical strain associated with the gel-to-sol transition of the hydrogel network. Additionally, dynamic strain tests are particularly important for assessing self-healing performance. Under low strain conditions, G′ > G″ indicates a stable gel state. Conversely, at a high strain, G′ < G″ indicates network disruption and flow under simulated injection shear. Upon reducing the strain, G′ should promptly recover and exceed G″, thereby confirming structural recovery [99]. For example, PEG-peptide/PEDOT:PSS hydrogels were shown to demonstrate characteristic self-healing properties during dynamic strain assessments, with G′ > G″ under a low strain. Under high strain, the hydrogel transitioned into a sol state, characterized by G″ > G′. Following the removal of strain, the hydrogel reverted to its gel state, although full recovery of the initial modulus may have required additional time [99].

### 4.3. Tissue Adhesion

Tissue adhesion is essential for the stable retention of ICHs within wet, dynamic, and irregular muscle defect sites. VML defects frequently bleed, produce exudate, and experience continuous muscle movement. Without sufficient adhesion, ICHs may migrate, detach, or develop interfacial gaps with host tissue. This compromises their local scaffold function and diminishes the efficiency of electrical coupling between the ICH and muscle, nerve, or vascular tissue. Consequently, tissue adhesion is a key requirement for transforming ICHs from pure injectable materials into multifunctional interfacial material. Furthermore, robust adhesion can significantly decrease interfacial resistance and enhance the stability of electrical stimulation and signal recordings [144]. The adhesion mechanisms within ICHs primarily encompass catechol- and dopamine-mediated adhesion [190], aldehyde-amine Schiff base reactions [191], electrostatic interactions [192], hydrogen bonding [190], multipoint physical entanglement [193], and topological adhesion [194]. Mussel-inspired catechol chemistry enables ICHs to adhere to wet tissue via multiple interactions, including hydrogen bonding, π–π stacking, metal coordination, and oxidative covalent bonding [195]. For example, Li et al. developed a tannic acid- and silicate ion-functionalized PVA-starch hydrogel, in which tannic acid formed abundant hydrogen bonds with the PVA and starch chains and interacted with silicate ions to construct a rapidly gelling adhesive network [196]. Aldehyde-containing hydrogels can form dynamic imine bonds with the amino groups on tissue proteins for rapid and reversible tissue adhesion, such as o-phthalaldehyde-based injectable hydrogel adhesives [197,198]. Electrostatic interactions arise from the attraction between charged polymer chains and oppositely charged tissue components, and hydrogen bonding contributes reversible interfacial binding through hydroxyl, carboxyl, amide, or phosphate groups [199]. In addition, physical entanglement and topological adhesion at multiple points strengthen the hydrogel–tissue interface by allowing polymer chains to interpenetrate or mechanically lock with hydrated tissue networks, as reported in studies on tough alginate–polyacrylamide or chitosan-mediated topological tissue adhesives [200,201].

Tissue adhesion of ICHs is commonly evaluated using lap–shear, tensile pull-off, peel, and burst–pressure tests [202]. Lap–shear tests quantify the shear adhesion strength on wet tissue, such as skin, muscle, liver, or heart, whereas tensile and peel tests assess normal adhesion strength and interfacial toughness, respectively [203]. Burst–pressure tests are particularly useful for evaluating sealing or hemostatic capabilities under fluid pressure. For conductive tissue interface hydrogels, adhesion tests should be further combined with electrical measurements, including impedance, conductivity retention, and signal stability under cyclic deformation, as stable adhesion directly determines the efficiency of electrical stimulation and bioelectrical signal recording [199]. For stimulation interfaces, charge storage capacity (CSC), derived from cyclic voltammetry, should be distinguished from pulse-based charge injection capacity (CIC), which depends on waveform, pulse width, and allowable electrode polarization [36]. ICAA exhibited a reported charge-injection density of 139 μC cm^−2^ under ±0.5 V, 1.5 ms biphasic pulses [157]. Because this value summed cathodic and anodic charges, it should not be directly equated with a per-phase safe injection limit.

### 4.4. Physiological Stability

Physiological stability of ICHs is critical for preserving their dimensional, mechanical, and electrical integrity in bodily fluids environments, thereby preventing interference with muscle contraction and neurovascular regeneration [188]. Upon exposure to bodily fluids, ICHs typically undergo water absorption with subsequent swelling. While moderate swelling can enhance nutrient exchange and cell infiltration, excessive swelling may cause volumetric expansion, reduce the modulus, dilute conductive networks, disrupt tissue-adhesive interfaces, and even compress newly formed blood vessels, nerves, and muscle fibers [188]. Physiological stability of ICHs is primarily achieved by regulating the network architecture and the balance between hydrophilic and hydrophobic components [188]. Enhancing the cross-linking density and developing double network or interpenetrating network structures may limit excessive bodily fluid uptake. For example, Gu et al. established a microphase semi-separated network through hydrogen bonding, electrostatic interactions, and chain entanglement among PVA, PEDOT, and PSS [204]. This configuration effectively interlocked the mechanical and conductive phases, thereby minimizing hysteresis and interfacial slippage while preserving optimal mechanical and electrical stability in wet environments. Moreover, the network stability can be further increased by incorporating rigid or semi-rigid elements, such as chitosan, cellulose, MXene, rGO, or nanoclay [205]. In addition, by integrating ionic liquids or hydrophobic domains and constructing zwitterionic or polyelectrolyte networks, the osmotic pressure, counterion migration, and water absorption may be further regulated, thereby enhancing the wet-state dimensional stability when exposed to bodily fluids [85]. Electrical stability should be assessed after equilibration in physiological media and during repeated electrical and mechanical loading. The injectable, conductive, adhesive, anti-swelling (ICAA) hydrogel exhibited a four-probe conductivity of 92.43 ± 7.65 S m^−1^ after full swelling in PBS. It retained an impedance of approximately 120 Ω at 1 kHz after four weeks in PBS at 37 °C and showed negligible change in charge injection over 10^5^ electrical cycles, with conductivity remaining approximately 95 S m^−1^ [157]. These findings demonstrate durability under the tested conditions; combined post-injection, cyclic-deformation, and in vivo evaluations remain necessary for skeletal muscle applications.

### 4.5. Biodegradability

Skeletal muscle regeneration encompasses several stages, including inflammation, proliferation, maturation, remodeling, and functional recovery, which can extend over several weeks or more [206]. Ideally, ICHs should offer temporary mechanical support, facilitate electrical signal transmission, and regulate the microenvironmental during the initial stages of regeneration, subsequently undergoing gradual degradation as newly formed myofibers, blood vessels, and nerves replace the hydrogel scaffold. Excessively rapid degradation may compromise the structural integrity and conductivity of ICHs. Conversely, if the ICH persists for too long, it may provoke foreign body reactions, fibrotic encapsulation, or chronic inflammation [207]. ICHs degrade through mechanisms such as hydrolysis [208], enzymatic degradation [209], redox-responsive cleavage [28], or dynamic covalent bond dissociation [210]. ICHs incorporating ester bonds, anhydride bonds, or hydrolysable PEG-acrylate structures typically undergo hydrolytic degradation [208]. In contrast, natural polymers, such as collagen, gelatin, hyaluronic acid, fibrin, and components of the extracellular matrix (ECM), are susceptible to enzymatic degradation in vivo [211]. Redox-responsive structures, including disulfide bonds, thioethers, selenoethers, and boronate esters, are sensitive to ROS or local redox conditions [28]. Additionally, Schiff base and acylhydrazone bonds allow the network to gradually disassemble through dynamic bond exchange or hydrolysis. Under physiological conditions, hydrolysis cleaves water-sensitive covalent bonds in cross-linkers or polymer backbones, thereby reducing network connectivity [208]. Enzymatic degradation involves substrate-specific cleavage, such as hyaluronidase-mediated degradation of hyaluronic acid and proteolytic cleavage of collagen or gelatin, with kinetics dependent on enzyme accessibility and network density [146,212]. Unlike these cleavage processes, reversible junction dissociation may cause network disassembly without polymer-chain degradation or complete clearance of conductive fillers [99,213]. In skeletal muscle injuries, particularly those complicated by diabetic or infected wounds, elevated levels of ROS and inflammatory enzymes can significantly influence ICH degradation kinetics. Therefore, the degradation behavior of ICHs should be systematically evaluated through both in vitro and in vivo studies. In vitro assays commonly examine mass loss, swelling ratio, gel fraction, morphology, rheological recovery, mechanical testing, and conductivity retention under PBS, enzymatic, or ROS-mimicking conditions. Molecular analyses such as Fourier-transform infrared spectroscopy (FTIR), nuclear magnetic resonance (NMR) spectroscopy, gel permeation chromatography (GPC), X-ray photoelectron spectroscopy (XPS), inductively coupled plasma mass spectrometry (ICP-MS), and liquid chromatography–mass spectrometry (LC-MS) can further identify bond cleavages, molecular weight changes, conductive filler release, metal ion release, and degradation products [213]. In vivo degradation is usually assessed by residual material volume, imaging or fluorescence tracking, histological staining, and inflammatory or fibrotic responses around the implanted hydrogel [212]. For example, MXene-functionalized ICHs, which were cross-linked through synergistic dynamic imine, acylhydrazone, and disulfide bonds, underwent progressive reconstruction and cleavage under physiological and redox conditions. Additionally, Nb_2_C MXene demonstrates H2O2- and enzymatic-responsive degradation [28]. Despite advances in ICHs, conductive component degradation continues to pose significant challenges. The in vivo degradation pathways and metabolic fate of materials such as PEDOT:PSS, rGO, CNTs, certain metallic nanomaterials, and some MXenes are inadequately understood [57]. After the ICH matrix degrades, conductive fillers may persist within the tissue. Specifically, in PEDOT:PSS systems, the potential release of PSS and its inherent acidity were shown to adversely impact long-term biocompatibility [214]. Natural polyanions, including hyaluronic acid [215], chondroitin sulfate [214], heparin [216], and dextran sulfate [217], have been investigated as alternatives or partial substitutes for PSS to mitigate these issues and enhance the biocompatibility and degradation safety of PEDOT-based ICHs.

### 4.6. Biocompatibility

Biocompatibility is essential for the use of ICHs in skeletal muscle repair. Given that ICHs are frequently administered into deep muscle tissue and may be positioned in proximity to nerves and blood vessels, their biocompatibility must extend beyond low cytotoxicity. Biocompatibility of ICHs should also include hemocompatibility, tissue compatibility, immune compatibility, the safety of degradation products, and the safety of long-term implantation [189]. Additionally, an optimal ICH should not only be non-toxic but should also actively facilitate the coordinated involvement of myogenic cells, vascular endothelial cells, nerve-associated cells, and immune cells [28]. For example, MXene-functionalized ICHs were reported to have an exceptionally low hemolysis rate, with no significant abnormalities observed in major organs or blood biochemical parameters, indicating favorable in vivo safety [218]. Furthermore, the ICHs exhibited excellent biocompatibility while also effectively scavenging ROS, promoting M2-like macrophage polarization, enhancing angiogenesis, and facilitating myogenic differentiation, highlighting their integrated biological compatibility and regenerative functionality [57]. F-rGO/PEG injectable conductive hydrogel electrodes have demonstrated excellent tissue compatibility. The ICHs effectively mitigated inflammation and signal instability, which are typically induced by mechanical mismatch, inadequate interfacial contact, and rigid stimulation [57]. Consequently, biocompatible ICHs must achieve an optimal balance among cytocompatibility, immune tolerance, mechanical congruence, electrical functionality, and long-term safety. Maintaining this balance is critical for regenerating skeletal muscle and restoring neuromuscular function.

Moreover, injectable hydrogel matrices can serve as cell- and protein-delivery depots for skeletal muscle repair. Microporous gelatin hydrogels improved mesenchymal stem cell engraftment, host–cell infiltration, myoblast differentiation, and muscle regeneration in a murine volumetric muscle loss model [219]. Likewise, injectable PEG–fibrinogen microspheres enabled localized, sustained Cripto release, increased Pax7-positive progenitors and myogenic markers, and accelerated tibialis anterior regeneration after acute injury [220]. Incorporating these functions into conductive hydrogels may couple electrical support with cellular and biochemical cues, although cell-compatible gelation and release kinetics require coordinated optimization. A comparative overview of the principal advantages and potential limitations associated with the multifunctional design of ICHs is provided in Table 3.

Summarizing, the success of ICHs in skeletal muscle repair is not solely dependent on their electrical conductivity; instead, it is the result of a synergistic interaction among multiple functional properties. The shear-thinning behavior of ICHs facilitates minimally invasive delivery into irregular defect sites, while their self-healing capacity contributes to maintaining network integrity and conductive continuity in mechanically dynamic environments. Furthermore, the tissue adhesion of ICHs is critical for establishing stable interfaces with the host tissue. ICH physiological stability is essential for preserving dimensional, electrical, and mechanical stability in vivo. Biodegradability is a key factor in aligning the functional lifespan of the material with the tissue regeneration process, and biocompatibility forms the fundamental basis for ensuring long-term safety and promoting regenerative activity. Moreover, cell and growth-factor delivery extends ICHs from electroactive scaffolds to localized platforms integrating electrical support with regenerative biological signaling.

## 5. Bioactive Mechanisms of Injectable Conductive Hydrogels

**Figure 5:**
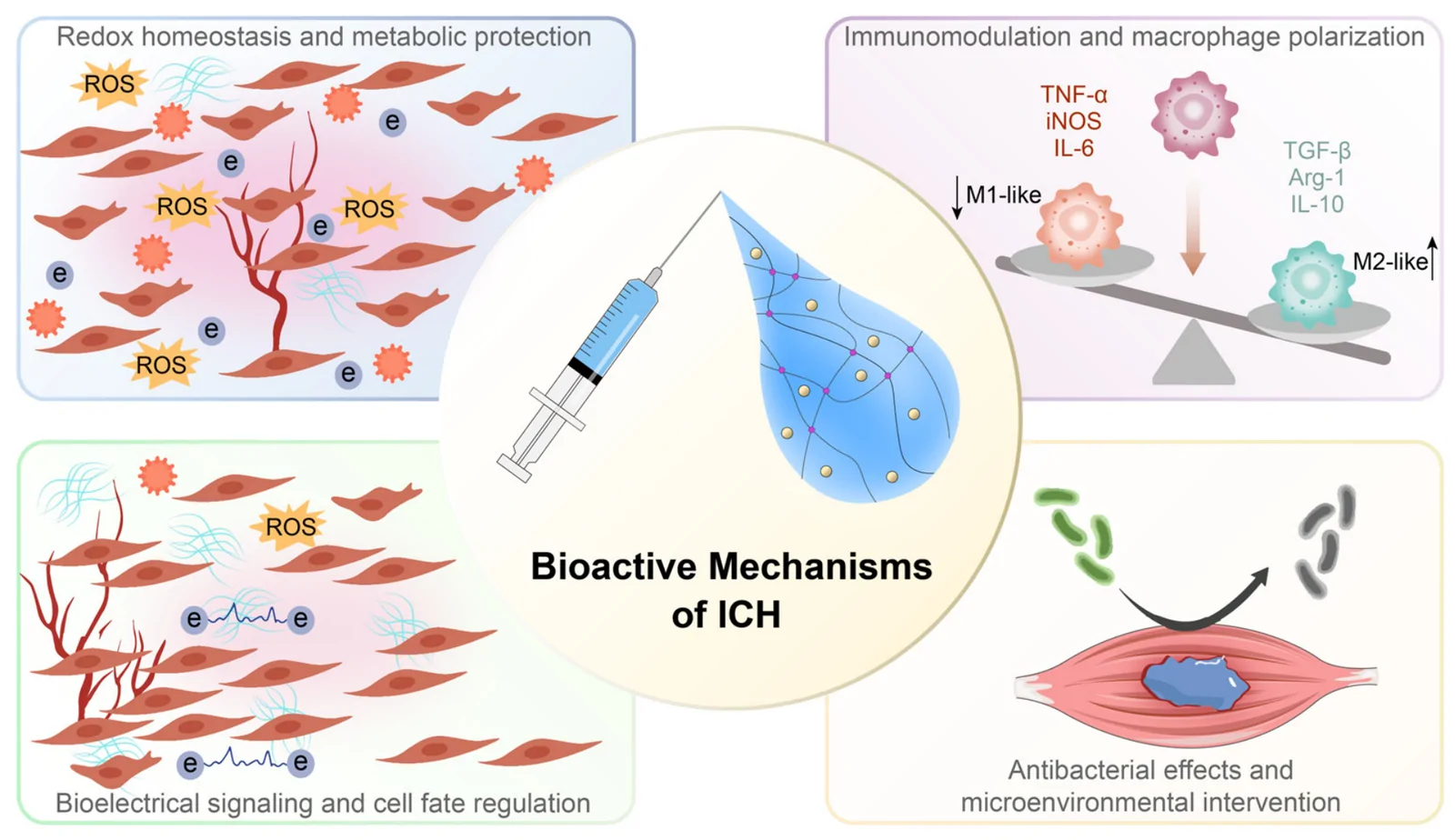
Principal bioactive mechanisms through which injectable conductive hydrogels support skeletal muscle repair. Redox-active components and electron-transfer pathways can scavenge excessive ROS, protect cellular metabolism, and restore redox homeostasis. Immunomodulatory cues can limit a prolonged M1-like inflammatory program characterized by tumor necrosis factor-α (TNF-α), inducible nitric oxide synthase (iNOS), and interleukin-6 (IL-6) while favoring a reparative M2-like program associated with transforming growth factor-β (TGF-β), arginase-1 (Arg-1), and interleukin-10 (IL-10). Conductive networks facilitate endogenous or externally applied bioelectrical signaling, thereby influencing membrane potential, Ca^2+^-dependent pathways, and myogenic cell-fate progression. Antibacterial components reduce microbial burden and biofilm-associated inflammation, indirectly preserving a regeneration-permissive microenvironment. Downward and upward arrows denote reduced and increased biological responses, respectively.

Skeletal muscle regeneration following injury relies on the ongoing remodeling of the local microenvironment [39]. Recent research has demonstrated that elevated oxidative stress, persistent inflammation, and disrupted endogenous bioelectrical signals at the injury site can significantly affect cell migration, proliferation, and differentiation, influencing the outcome of skeletal muscle regeneration [227]. However, many traditional biomaterials primarily provide structural support or serve as defect fillers, with a limited capacity to modulate microenvironmental factors, thus constraining their ability to precisely control the regeneration process [228]. With advances in biomaterials for regenerative medicine, ICHs have attracted increasing attention as promising platforms for skeletal muscle regeneration. These materials are engineered by incorporating conductive elements into dynamically tunable polymer networks [229]. Upon injection, ICHs form electroactive interfaces in situ that conform to the architecture of injured tissue [230]. Furthermore, their charge transport, inflammation regulation, and electrical signal transmission capabilities enable ICHs to modulate cellular behavior and immune responses [230]. As summarized in Figure 5, we examine the role of ICHs in skeletal muscle repair from the perspectives of injury-microenvironment remodeling and cellular regulation. Four key aspects are considered: redox homeostasis, immune regulation [13], bioelectrical signaling [218], and the modulation of infection-associated inflammatory microenvironments [231]. Where direct skeletal-muscle evidence is limited, studies from other tissues are cited only to illustrate transferable material or mechanistic principles and are not interpreted as direct evidence of skeletal muscle regeneration.

### 5.1. Redox Homeostasis and Metabolic Protection

Oxidative stress is recognized as a critical pathological factor within the microenvironment of injured tissue. When ROS production exceeds the buffering capacity of injured tissue, oxidative injury spreads from membrane lipid oxidation to protein dysfunction and genomic instability [232]. These processes compromise cellular function and sustain chronic inflammation, impeding tissue regeneration. Consequently, regenerative biomaterial development has increasingly focused on modulating ROS levels through material systems to improve the injury microenvironment [233]. Within damaged skeletal muscle, ROS dysregulation, in addition to being cytotoxic, reshapes the trajectory of regeneration by disturbing satellite cell activity, mitochondrial metabolism, myogenic progression, and myofiber maturation [234]. Furthermore, changes in ROS levels influence not only cellular damage but also redox-sensitive signaling pathways involved in skeletal muscle regeneration. Excessive or persistent ROS accumulation may impair satellite cell activity, mitochondrial metabolism, and myogenic progression [233]. Among these pathways, the Nrf2–Keap1 signaling axis is recognized as a pivotal regulatory node [235]. Under normal conditions, Keap1 associates with Nrf2 to promote its degradation. However, in response to changes in the redox environment, oxidative stress loosens the Keap1-mediated sequestration of Nrf2, causing nuclear Nrf2 to induce ARE-centered antioxidant processes involving HO-1, SOD, CAT, and NQO1 [236]. These molecular changes contribute to restoring the intracellular redox state toward equilibrium while limiting inflammation-driven secondary injury. In damaged muscle, Nrf2 activation is meaningful as it links antioxidant defenses with metabolic reprogramming. This connection may allow satellite cells to shift from stress adaptation toward activation, differentiation, and myofiber reconstruction [235].

Reported ICHs achieve antioxidant activity through chemically defined redox-active or catalytic components rather than through electrical conductivity alone [237]. Common conductive components, such as PPy [238], PANI [239], and graphene derivatives [239], exhibit reversible electron transfer capabilities. These materials can function as electron donors or acceptors in redox reactions to scavenge highly reactive oxygen-derived species, particularly •OH and O2•−. Electron transfer occurs on the surface of material, potentially mitigating local oxidative pressure and partially restoring redox homeostasis within the damaged microenvironment [240]. For example, Lee et al. provided a representative example by using graphene oxide/alginate microgels as a ROS-buffering interface. In this system, the graphene-derived component enhanced cellular tolerance to oxidative stress and mitigated niche deterioration caused by ischemia. Additionally, polymer networks generated with dynamic or redox-adaptive reversible linkages, including imine-type dynamic bonds [182] and Schiff-base bonds redox-responsive chemistry [241], confer hydrogels with adaptive responsiveness to oxidative conditions. In environments with elevated ROS levels, reversible chemical bonds can be broken and reformed, a dynamic process that may alleviate the oxidative burden at the molecular level and maintain network integrity [182]. For example, Peng et al. developed an injectable HCS-TBA@Py conductive hydrogel, in which hydroxypropyl chitosan was coupled with 2,3,4-trihydroxybenzaldehyde through imine chemistry, whereas in situ pyrrole polymerization introduced electroconductive domains into the hydrogel network. This dynamically cross-linked system exhibited self-healing and antioxidant properties, illustrating how adaptive bonds can help preserve network integrity while alleviating oxidative injury. Additionally, significant efforts have been directed toward developing multilevel antioxidant systems by incorporating antioxidant peptides [242], enzyme-mimicking nanoparticles [243], or other ROS-scavenging components [244] into ICHs. Within this design framework, the material may directly neutralize some of the ROS and simultaneously activate endogenous antioxidant pathways within cells to neutralize more ROS [245]. Collectively, these interactions establish a synergistic antioxidant mechanism predicated on a paradigm in which material regulation generates a cellular response [246]. This redox-regulatory approach has the potential to provide stable and enduring microenvironmental interventions for chronic inflammatory diseases and non-healing wounds. However, it is important to recognize that ROS scavenging does not inherently result in regenerative benefits. In the context of skeletal muscle repair, the critical consideration is whether the material can effectively diminish oxidative stress while preserving essential signaling activity and supporting normal regenerative processes [247].

### 5.2. Immunomodulation and Macrophage Polarization

Tissue regeneration is predominantly governed by the dynamic equilibrium of the immune microenvironment. Macrophage polarization is recognized as a key immune event influencing the trajectory of tissue repair [248]. Early inflammatory macrophages support debridement and pathogen control of injured muscle while releasing TNF-α and IL-1β to initiate downstream immune signaling. During the subsequent repair phase, macrophages shift toward the anti-inflammatory and pro-regenerative M2-like phenotype, facilitating angiogenesis, cell proliferation, and matrix remodeling [249]. In skeletal muscle regeneration, macrophage polarization can be assessed by the static ratio between pro-inflammatory and reparative phenotypes [250]. However, of greater importance is the timely transition from the inflammatory clearance phase to the regenerative phase. Delayed macrophage switching can trap the lesion in a pro-inflammatory state, thereby limiting satellite cell expansion, vascular ingrowth, and constructive matrix remodeling [251].

ICHs can form conductive pathways within dynamic polymer networks that help couple electrical signals, ion migration, and bioactive molecule delivery [252]. Upon exposure to external electrical stimulation or endogenous bioelectrical signals, electrical cues can propagate through ICHs. These cues may be sensed through membrane-associated ion channels and calcium-handling mechanisms, leading to early electrophysiological or calcium-dependent responses, such as the influx of Ca^2+^ [253]. Changes in Ca^2+^ concentrations significantly affect the various pathways associated with macrophage polarization [254]. For example, Ca^2+^-dependent signaling can modulate the activation state of NF-κB, with attenuated NF-κB-driven inflammatory signaling typically correlating with the reduced expression of inflammatory cytokines [254]. Changes in the electrochemical microenvironment induced by ICHs may be associated with macrophage-regulatory pathways, including STAT6- and PI3K/Akt-related signaling, although direct causal evidence in skeletal muscle ICH systems remains limited. These pathways are pivotal in M2-like macrophage differentiation [222]. STAT6 activation promotes a reparative macrophage program marked by Arg-1 and CD206, and PI3K/Akt signaling upregulates anti-inflammatory cytokines and genes related to tissue repair. For example, macrophages cultured on conductive interfaces exhibited upregulated M2-like markers and decreased inflammatory cytokine secretion [255]. Immunomodulatory factors and other anti-inflammatory molecules can be incorporated into ICHs to further guide the macrophage phenotype transition [256]. When combined with electrical modulation, these systems may shift the local immune milieu toward a repair-associated state [222]. In addition, immunomodulatory ICHs can be engineered with sequential release characteristics to mitigate excessive inflammation during the initial repair phase and promote tissue remodeling in subsequent stages. These ICH designs offer innovative regulatory approaches for promoting the regeneration of complex tissues [257].

### 5.3. Bioelectrical Signaling and Cell Fate Regulation

Continuous and semi-continuous conductive pathways within ICHs provide a stable conduit for electrical signal transmission at the material–cell interface. With their electroactive architecture, ICHs provide structural support while shaping key cellular responses at the material–cell interface, including cell proliferation, motility, and lineage commitment [258]. This function has great significance in skeletal muscle regeneration. As a prototypical excitable tissue, successful skeletal muscle repair requires not only the generation of new tissue mass but also the restoration of excitation conduction, neuromuscular coupling, and myofiber maturation [218]. Consequently, the relevance of bioelectrical signals extends beyond cellular activation, and is crucially determined by the extent to which these signals facilitate myofiber maturation, neuromuscular coupling, and the continuity of local excitation conduction [217].

When combined with external electrical stimulation, ICHs can facilitate the transmission of electrical cues throughout tissue microenvironment [218]. These signals are detected by cells via voltage-responsive ion channels at the plasma membrane. Notably, the opening of Ca^2+^-permeable channels is regarded as a critical indicator of successful electrical signal transduction. A Ca^2+^ influx modulates the membrane potential and can initiate a cascade of downstream signaling pathways, which can ultimately influence cytoskeletal remodeling and the expression of related genes [259]. Furthermore, a Ca^2+^ influx induced by electrical stimulation can engage MAPK/ERK signaling, a pivotal regulator of cell migration, proliferation, and cell cycle-associated transcriptional programs [260]. In addition, electrical signals have the potential to increase pro-angiogenic signaling, particularly VEGF-associated responses, through the PI3K/Akt signaling axis [261]. Electrical stimulation has also been proposed to interact with YAP/TAZ-related mechano-transduction pathways, thereby contributing to myogenic differentiation regulation, myofiber maturation, improvement of local blood supply, and subsequent neuromuscular functional integration [262].

In addition to external electrical stimulation, ICHs can interact with endogenous bioelectrical activity in the body. In skeletal muscle injury, a stable interface established in the injured region may enhance the electrical responsiveness of local cells and have additional significant implications. The interface may also create a more conducive electrophysiological environment for reconstructing neuromuscular junctions, synchronizing myofiber excitation, and restoring excitation–contraction coupling [263]. For example, a conductive MXene/adECM matrix illustrated how electroactive hydrogels may rebuild the electrophysiological niche. When applied to VML defects, the material improved calcium signaling, neuromuscular junction formation, reinnervation, and nerve stimulation-induced contraction, supporting the concept that conductive hydrogels can help rebuild an electrophysiological interface that promotes functional skeletal muscle regeneration.

Researchers are currently endeavoring to integrate piezoelectric and mechanical-to-electrical conversion units into hydrogel systems. These units enable ICHs to transduce mechanical stimuli arising from tissue movement into localized electrical signals [264]. This mechanism partially aligns with the physiological characteristics of skeletal muscle, which undergoes prolonged stretching, contraction, and recurrent mechanical perturbations. Consequently, it is theoretically appropriate to develop bioelectronic interfaces that can dynamically respond to skeletal muscle movement. ICHs that support this mechano-electrical coupling have the potential to provide a relatively stable electrophysiological regulatory environment without the need for continuous external electrical stimulation. In this manner, these systems may contribute to the sustained modulation of cellular electrical signaling [265].

### 5.4. Antibacterial Effects and Microenvironmental Intervention

In contrast to redox, immune, and bioelectrical regulation, infection-related intervention should be recognized as a crucial function of ICHs in managing complex wounds or contaminated injuries, because infection can perpetuate inflammation, oxidative stress, and matrix disorganization [266,267]. In complex skeletal muscle injuries, infection is a significant upstream factor that exacerbates the progressive deterioration of the local microenvironment [268]. Persistent bacterial colonization and biofilm formation may continuously stimulate innate immune sensors, particularly TLR-associated pathways, which converge on NF-κB and MAPK signaling to amplify and sustain inflammation [269]. Consequently, neutrophils and pro-inflammatory macrophages may persist in the affected area, causing sustained production of cytokines, including TNF-α, IL-1β, and IL-6 [270]. Bacterial virulence factors, proteases, and limited diffusion can exacerbate matrix degradation, local hypoxia, and oxidative stress. These factors may disrupt mitochondrial function, impair satellite cell activation and myogenic differentiation, and increase the likelihood of normal repair processes transitioning toward fibrotic replacement [267]. From this perspective, the detrimental effects of infection extend beyond the burden of the pathogen itself. More importantly, infection can reinforce the pathological coupling among prolonged inflammation, redox imbalance, and matrix disorganization to hinder the progression of the injured microenvironment toward a regenerative state [271]. Infection therefore blocks repair through several mechanisms: it slows necrotic debris clearance, weakens satellite cell activation, disrupts myogenic differentiation, and increases the probability of fibrotic replacement [267].

The primary significance of ICHs is their ability to incorporate pathogen control within a localized regenerative interface. In situ gelation enables ICHs to adapt to irregularly shaped injury sites, thereby retaining and exposing antibacterial drugs, antimicrobial peptides, and other active agents throughout the injury site [272]. For example, Ren et al. developed a conductive GelMA/Ti_3_C_2_ hydrogel dressing loaded with collagen-binding antimicrobial peptides (V-Os) and showed that the modified peptides were closely bound to the hydrogel matrix, enabling prolonged local antibacterial activity. Furthermore, by incorporating conductive elements into the material–bacteria interface transformed the interface from a passive contact surface to an active agent [273]. The charge distribution, ion exchange, and interfacial electrochemical properties of an ICH can disrupt bacterial membrane potential homeostasis. For example, Xie et al. constructed a polyphenol conductive nanozyme-enhanced redox hydrogel containing PDA-Fe-PEDOT nanozymes, in which PEDOT mediated interfacial electron transfer, which sustained dopamine redox cycling and promoted H2O2 to ·OH conversion. Notably, antibacterial ROS generation should be distinguished from uncontrolled oxidative stress in regenerative tissues. Ideally, antibacterial activity should be spatially confined to the material–bacteria interface while avoiding excessive ROS accumulation in host cells. This converted the hydrogel–bacteria interface into an active antibacterial microenvironment [274].

In summary, the immunomodulatory, redox-buffering, myogenic-inductive, and electrophysiological supportive properties of ICHs are more likely to confer tangible regenerative benefits only when pathogen-induced inflammatory amplification and redox imbalances are mitigated. These microenvironmental requirements do not alter the primary mechanistic framework previously discussed; instead, they explain why, under specific pathological conditions, ICHs should simultaneously integrate pathogen control and regenerative interface stabilization within a single material system. In complex skeletal muscle injuries, antibacterial intervention should not be viewed only as pathogen removal; its greater value lies in preventing infection from locking the lesion into a non-regenerative inflammatory state.

## 6. Applications of Injectable Conductive Hydrogels in Skeletal Muscle Regeneration

Skeletal muscle regeneration is a temporally ordered process involving inflammation resolution, myogenic progression, vascularization, reinnervation, and functional integration [6,275]. Because reported conductivity values of ICHs are highly dependent on the measurement method and testing conditions, their relevance to skeletal muscle repair should not be judged by absolute conductivity alone. Instead, the key requirement is whether the conductive network can maintain stable electrical coupling under hydrated and mechanically dynamic conditions, thereby supporting the transmission of endogenous or externally applied electrical cues across the injured muscle while remaining compatible with the surrounding tissue. In the initial phase post-injury, the outcome largely depends on the timely management of secondary inflammation and oxidative damage [276,277]. As the process transitions into the regenerative phase, the critical focus becomes successful satellite cell activation, myogenic differentiation, and tissue reorganization [278,279]. In the subsequent stages, angiogenesis, reinnervation, and the functional integration of newly formed muscle tissue become the pivotal processes determining the transition from tissue filling to genuine functional recovery. These stage-dependent regenerative requirements have driven the development of ICHs with diverse cross-linking strategies, conductive media, physiological stability, and bioactive functions. As summarized in Table 4, representative ICH systems have been engineered not only to promote myogenic differentiation, angiogenesis, innervation, and microenvironmental remodeling, but also to serve as injectable electromyographic electrodes and neuromodulation interfaces. These examples indicate that the effectiveness of ICHs is determined not by conductivity alone, but by the coordinated integration of network architecture, electrical performance, physiological stability, and biological activity to meet specific therapeutic demands.

**Figure 6:**
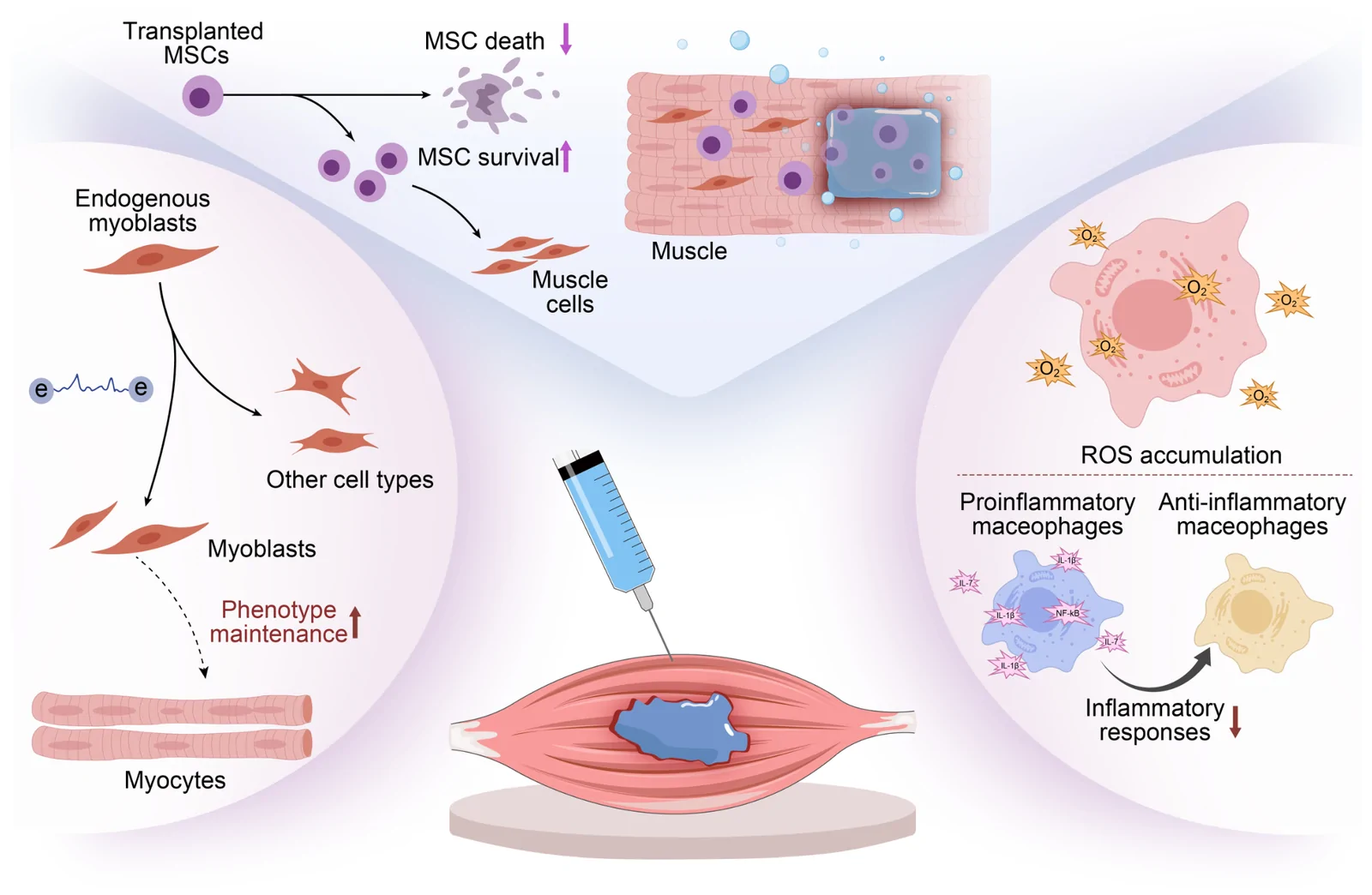
Hierarchical therapeutic roles of injectable conductive hydrogels in skeletal muscle regeneration. Following minimally invasive delivery into an irregular defect, the hydrogel first acts as a local matrix that retains transplanted mesenchymal stem cells (MSCs), improves their survival, and supports their differentiation toward muscle-lineage cells. The electroactive matrix can simultaneously preserve endogenous myoblast phenotype, promote myoblast differentiation and fusion into myocytes, and provide conductive cues for tissue organization. At the microenvironmental level, ROS-scavenging activity reduces oxidative stress, while immunomodulatory signals facilitate the transition from persistent pro-inflammatory macrophage responses toward repair-associated macrophage phenotypes. The integration of cell delivery, redox regulation, immune reprogramming, and bioelectrical coupling shifts ICHs from passive defect-filling scaffolds to active soft interfaces that guide structural and functional muscle regeneration.

Taken together, these representative applications demonstrate that ICHs should be regarded not merely as injectable materials with electrical conductivity, but as dynamic interfaces that actively participate in skeletal muscle reorganization [4]. Accordingly, the central consideration in ICH design is not the number of functions incorporated into a material, but whether these functions can be effectively coordinated within the specific context of skeletal muscle injury to establish a hierarchical intervention pathway [4]. As illustrated in Figure 6, ICHs can initially serve as local delivery matrices that enhance the retention and survival of transplanted mesenchymal stem cells and other therapeutic payloads within the defect region [160]. They can subsequently regulate the host microenvironment by alleviating oxidative stress, modulating macrophage responses, and preserving the myogenic phenotype of endogenous cells [95]. At a more advanced level, their conductive networks can support myoblast maturation, electrical signal transmission, and tissue organization, thereby promoting the transition from localized therapeutic scaffolds to three-dimensional bioelectronic interfaces [218]. Therefore, the evolution of ICHs represents not merely an expansion of material functions, but a shift from passively supporting repair to actively shaping the regenerative trajectory [4].

### 6.1. ICHs Deliver Therapeutic Agents for Skeletal Muscle Regeneration

Injured skeletal muscle is frequently characterized by increased inflammatory cytokine levels, oxidative overload, high proteolytic activity, and extracellular matrix destabilization [280,281]. When exogenous therapeutic agents are introduced into this type of environment, they are susceptible to rapid degradation, inactivation, and limited duration of efficacy [281]. ICHs can serve as local delivery systems to encapsulate and release stem cells, myogenic progenitor cells, growth factors, small-molecule drugs, and other bioactive components [282]. The in situ gelation ability of ICHs forms three-dimensional networks that conform to irregular defect sites, increasing the local retention of therapeutic components and prolonging their effective duration of action [283].

Cell-laden ICH matrices and growth-factor-releasing ICHs represent complementary delivery strategies for skeletal muscle regeneration. For cell delivery, injectable matrices must protect cells from shear stress during injection while maintaining cell viability, adhesion, proliferation, and myogenic differentiation after implantation. Zhang et al. developed injectable gelatin/rGO conductive microcryogels loaded with muscle-derived stem cells. The porous microcryogels protected the encapsulated cells during injection, enhanced their proliferation and myogenic differentiation, and increased new myofiber and blood vessel formation in a murine VML model [9]. In parallel, conductive hydrogels can serve as local depots for growth factors whose short half-lives and rapid diffusion otherwise limit therapeutic efficacy. Zhao et al. designed biodegradable conductive anisotropic IPN cryogels capable of sequentially delivering IGF-1 and VEGF. The temporally coordinated release supported myogenic differentiation and vascularization and improved VML repair in vivo [10]. These studies indicate that cell and growth-factor delivery should be coordinated with hydrogel porosity, mass transport, degradation, release kinetics, conductivity, and injectability rather than treated as independent functions.

Unlike traditional inert hydrogels, ICHs with their conductive components can perform loading functions while facilitating charge transport, ion exchange, and redox-buffering [277]. Consequently, ICHs are dynamic and regenerative interfaces capable of continuous interaction with cells and the surrounding microenvironment. In terms of cell delivery, this implies that transplanted cells, when encapsulated within the ICH matrix, are surrounded by an electroactive microenvironment that may enhance cell attachment, morphological extension, and lineage progression [160]. This concept was demonstrated using a hyaluronic acid-based conductive self-healing matrix, in which the electroactive network provided a permissive 3D niche for C2C12 myoblasts and hADSCs that promoted cell delivery, adhesion-related behavior, and myogenic differentiation. In addition, the local conductive network within ICHs can be designed to modulate cellular activity and respond to electrical cues [218]. For example, engaging Ca^2+^-dependent signaling pathways and related myogenic transcriptional programs may help induce cells to transition from a survival state toward differentiation and fusion [15]. Given that CaMK, calcineurin/NFAT, MAPK/ERK, and PI3K/Akt signaling pathways are closely involved in myogenic commitment and differentiation-associated transcriptional regulation, the electroactive microenvironment of the ICH may enhance the activation of critical myogenic programs, such as MyoD, MyoG, and MEF2 [284]. Therefore, the principal role of ICHs is to protect, enhance, and locally organize these components, thereby aligning with the goal of improving therapeutic delivery. Unlike conventional delivery systems, however, ICH-based delivery incorporates local electrophysiological regulation and microenvironment adaptation.

### 6.2. ICHs Modulate the Pathological Microenvironment for Skeletal Muscle Regeneration

As research progresses, the therapeutic focus of ICHs in skeletal muscle repair has been significantly transformed. The current research focus extends beyond enhancing exogenous cells or bioactive factors and has shifted to whether ICHs themselves can directly modulate the tissue state post-injury [65,285]. In cases of severe skeletal muscle injury, repair failure is typically caused by more than a deficiency in pro-regenerative signals and instead generally results from the tissue persisting in a pathological steady state that does not support the initiation and progression of regeneration. In severe muscle injury, necrotic myofibers and a disrupted ECM maintain a pathological loop of inflammatory infiltration, oxidative stress, fibroblast activation, and impaired myogenic progression [286,287]. In essence, the ICH delivery layer primarily focuses on the mechanisms by which therapeutic agents are introduced into the lesion. Conversely, the modulatory layer addresses a more profound issue: whether the lesion possesses the requisite conditions to support its transition toward a regeneration-permissive state [2].

When considering redox regulation within the injured niche, the objective should be on mitigating the persistent inhibitory effect of oxidative stress on the regenerative program [235]. The antioxidant capacity of the ICH should be evaluated by whether it restores a regeneration-permissive redox state, rather than by ROS elimination alone [235]. This interfacial interaction can mitigate, to some extent, the ongoing damage inflicted by excessive ROS on cells and the ECM [238]. This approach is exemplified by MnO2@PD-HGC, an injectable conductive hydrogel designed to respond to the injured muscle microenvironment. Its ROS-scavenging activity reduced the oxidative burden in lesions and promoted skeletal muscle regeneration in vivo [95]. However, this process should not be construed as a straightforward cause-and-effect relationship; rather, by incorporating conductive materials into the ICH, the local redox balance was modulated to create a comparatively more stable metabolic environment conducive to regeneration.

From an immune standpoint, it is insufficient to categorize ICHs as only anti-inflammatory. ICH-mediated immune regulation can extend beyond simply suppressing inflammation. In skeletal muscle repair, the key task is to guide inflammation from an early clearance phase toward a repair-oriented immune state [288]. Effective skeletal muscle regeneration requires a well-regulated immune transition. Initially, a moderate pro-inflammatory response is required to remove necrotic debris and trigger signaling events that prepare the tissue for reconstruction [289]. Subsequently, this response must transition in a timely manner toward a repair-oriented state to facilitate satellite cell proliferation, angiogenesis, and matrix remodeling [290]. Prolonged delays during this transition may result in persistently elevated levels of inflammatory factors, which can hinder myogenic differentiation and predispose the tissue to fibrosis [267]. Therefore, prematurely suppressing inflammation may not be advantageous. It is crucial to permit the resolution of inflammation at an appropriate juncture, as this timing is intrinsically linked to the successful expansion of satellite cells and subsequent myogenic differentiation [289]. The conductive networks within ICHs can influence local immune cell phenotypes and cytokine secretion profiles to reshape the interfacial immune environment [218]. A mechanically compliant bioelectronic hydrogel used to treat diabetic VML further supports this concept. Immune-targeting polysaccharide motifs were combined with conductive PPy and gelatin to form an immunoregulatory interface that attenuated chronic inflammation and improved regeneration in a metabolically compromised repair environment.

In terms of cellular behavior and electrophysiological regulation, the conductive network within ICHs can improve the local potential distribution and signal transmission conditions, potentially increasing the responsiveness of myogenic cells to electrophysiological stimulation [218]. Skeletal muscle cell fate determination is intricately linked to the establishment of an excitable membrane [218]. Processes such as membrane depolarization, Ca2+ oscillations, cytoskeletal remodeling, and associated mechano-transduction processes are integral to the transition of these cells from proliferation to fusion and maturation. The conductive microenvironment within ICHs may enhance Ca2+-related signaling and improve local mechano-electrical coupling conditions to facilitate the progression of cells from initial survival to effective integration and maturation [218]. For example, Wu et al. reported that exercise-mimicking bioelectrical stimulation generated by a piezoelectric system synergized with anisotropic topographical cues to enhance myogenic differentiation via the Ca2+/CaN/NFAT2 pathway, which promoted myofiber alignment and functional muscle repair [291]. A critical consideration is that skeletal muscle repair is not generally limited by a deficiency in cells but instead by the absence of an environment that guides cells toward the appropriate fate [292]. Nonetheless, the primary focus in terms of cellular regulation is the redirection of the fate trajectory, which should not be equated with functional reconstruction itself [293].

Overall, the modulatory role of ICHs should be understood as pathological node reprogramming. Compared with delivery-enhanced therapy, which mainly improves the localization and persistence of therapeutic agents, ICH-dominated modulation aims to reshape the interdependence among oxidative stress, immune persistence, impaired myogenic progression, ECM disorganization, and electrophysiological discontinuity. By modulating these interconnected pathological processes, ICHs may redirect the injured tissue toward a regeneration-permissive trajectory. This distinction explains why ICHs act as active regulators of the regenerative trajectory in addition to being carriers.

### 6.3. Synergistic Effects of ICHs and Electrical Stimulation for Skeletal Muscle Regeneration

Microenvironmental remodeling can significantly improve the initial conditions for skeletal muscle regeneration. However, because skeletal muscle is an electrically excitable and contractile tissue, structural restoration does not inherently lead to functional recovery [294]. Even if newly formed tissue fills the defect area, the regenerated tissue may remain in a state of morphological repair with poor function and insufficient reinnervation, incomplete neuro-muscular junction reconstruction, limited membrane excitability, or disrupted local electrical conduction. In essence, although muscle-like tissue may form, it may not restore muscle function [295]. This represents a fundamental challenge that regenerative systems have yet to overcome. In this context, skeletal muscle is distinct from most non-excitable soft tissue. The endpoint of skeletal muscle repair should therefore be defined not by tissue occupancy alone but by the regeneration of an electrically excitable and contractile functional unit [296].

External electrical stimulation is extensively used to promote skeletal muscle cell maturation and functional training. It is also used to enhance the architectural organization and contractile competence of newly regenerated muscle [297]. However, in muscle injuries, the electric field is frequently attenuated and unevenly distributed due to factors such as scar formation, an increased local impedance, and tissue discontinuity [284]. Consequently, it is challenging to effectively deliver stimulation to the deeper regions of injured tissue. ICHs, by forming a flexible, continuous, and tissue-adaptive conductive network within the lesion, have the potential to spatially redistribute external electrical signals [4,218]. This redistribution may reduce the formation of functionally isolated regions caused by local conduction interruptions, exposing a greater number of cells to an electrophysiological regulatory range in which they can sense and respond to electrical cues [184].

Mechanistically, ICH-based bioelectronic interfaces exhibit at least three interrelated capabilities. First, the conductive network within ICHs enhances the efficiency with which external stimuli and local endogenous electrical activity modulate the cell membrane potential [218]. This enhancement may help open voltage-gated Ca2+ channels, enhance the intracellular Ca2+ influx and oscillations, and further amplify signaling pathways associated with myogenic differentiation and metabolic activation, including the CaMK, calcineurin/NFAT, MAPK/ERK, and PI3K/Akt pathways [297]. These changes may promote myoblast fusion and myofiber maturation. Second, more uniform electric-field propagation through ICHs may facilitate endothelial motility and increase pro-angiogenic signaling [298,299]. Upregulated pro-angiogenic signaling can enhance perfusion in the regenerative region, thereby sustaining nutritional and metabolic support for newly formed tissue. Third, ICHs may serve as local signal-coupling bridges to compensate for peripheral nerve injury or neuromuscular junction disruption. This may improve the electrical conduction conditions between nerve terminals and newly formed muscle tissue, creating a more favorable interfacial environment for neuromuscular junction reconstruction and functional integration [295,299,300].

Summarizing, future advancements in ICHs with enhanced translational potential are unlikely to be based on simple homogeneous conductive systems; instead, they will require sophisticated designs that incorporate spatially distributed conductive networks, anisotropic alignment, strain-responsive behavior, and compatibility with external stimulation parameters. Future studies should systematically optimize stimulation parameters, including current intensity, voltage amplitude, frequency, pulse duration, stimulation duration, and waveform, because inappropriate stimulation may induce cell stress, excessive contraction, or inflammatory responses. The full potential of ICHs as bioelectronic therapeutic platforms can only be realized when they can simultaneously adapt to the oriented skeletal muscle structure, dynamic mechanical environment, and electrophysiological propagation characteristics. Consequently, beyond facilitating tissue growth, the ultimate goal of ICHs is to maximize the restoration of functional properties in newly formed skeletal muscle tissue, including its excitability, integration, and contractility.

## 7. Conclusions and Outlook

In this review, we examined the design considerations for developing ICHs, which serve as innovative platforms for repairing and regenerating skeletal muscle. Unlike conventional hydrogels, which primarily provide structural filling or passive scaffold support, ICHs integrate minimally invasive injectability, electrical conductivity, dynamic network reformation, and microenvironment regulation within a single material system. This integrated functionality is particularly important for severe skeletal muscle injuries such as VML. Beyond tissue loss, these injuries are characterized by chronic inflammation, oxidative stress, inadequate vascularization, impaired reinnervation, and disrupted electrical signal transmission, which collectively compromise functional recovery.

Electronically conductive systems, including conductive polymers, carbon-based nanomaterials, MXenes, and metallic elements, promote efficient charge transport and generate low-impedance electrical contacts. Conversely, ionically conductive materials, such as free ions, ionic liquids, and ionic polymers, can replicate the ion-based electrophysiological milieu of native muscle tissue. Mixed ionic–electronic conductive systems are especially advantageous due to their ability to interface with electron- and ion-mediated processes produced externally and internally within the body, respectively. Hydrogels that are physically cross-linked can undergo reversible network disruption and reformation to support shear-thinning injectability and self-healing. In contrast, chemically cross-linked networks have high stability and biodegradability. Furthermore, physicochemical cross-linking integrates dynamic physical interactions, covalent or coordination bonds, and conductive phases, synergizing their functions.

In skeletal muscle repair, it is imperative to evaluate ICH properties through a comprehensive, multi-dimensional approach. Shear thinning is a critical property that determines the injectability of ICHs and therefore their ability to fill irregular defects. The self-healing capability of ICHs is essential for maintaining structural and electrical continuity during dynamic muscle tissue contractions. Tissue adhesive properties are crucial for ensuring seamless contact with host muscle tissue. The stability of ICHs is vital for preserving dimensional and electrical stability under moist physiological conditions. The material must also biodegrade to match the natural regeneration process. Biocompatibility and safety are foundational for ensuring immune compatibility and promoting regenerative properties.

Furthermore, bioactive regulation represents a significant research avenue in the study of ICHs. By incorporating conductive fillers and forming dynamic polymer networks, ICHs have the potential to participate in redox buffering, macrophage polarization, antibacterial treatment, and electrophysiological activity. Consequently, in addition to serving as scaffolds for cells, drugs, or growth factors, ICHs are active agents capable of dynamically modulating the pathological microenvironment and directing the repair process. By regulating ROS levels, the inflammatory cycle, cell membrane excitability, calcium flux, and pathways promoting angiogenesis and myogenesis, ICHs can help transition skeletal muscle injury from a detrimental state to a repair-conducive state. The rationale for using ICHs in skeletal muscle regeneration is currently undergoing a significant transformation. Superficially, ICHs function as delivery platforms that enhance the retention and activity of therapeutic cells, drugs, and molecules. More fundamentally, they act as regulatory interfaces that modulate oxidative stress, inflammation, immune phenotype shifts, and cell fate decisions. Additionally, they can function as bioelectronic interfaces that regulate electrical signal propagation, facilitate motor nerve–muscle interactions, and restore myofiber excitability, integration, and contractility. This evolution in understanding indicates a shift in material design objectives from promoting tissue regeneration to fully achieving functional tissue reconstruction.

Despite the advancements achieved thus far, certain challenges that remain must be resolved for the successful application of ICHs in skeletal muscle repair. From a clinical translation perspective, the following challenges are ranked by their relevance to safe and reproducible VML therapy.

Long-term biosafety and conductive-filler clearance. It is imperative to assess the long-term in vivo biocompatibility and biodegradability of conductive fillers. Materials such as conductive polymers, carbon nanomaterials, MXenes, and metallic fillers may persist after the ICH matrix degrades. There is currently only a limited understanding of their biodistribution, clearance, potential to induce inflammation, and the nature of their degradation byproducts. Future studies should develop conductive phases with defined degradation and clearance pathways.

Manufacturability, sterilization, and batch consistency. To support clinical translation, the manufacturability, sterilization, stability, injectability, batch-to-batch consistency, and safety of ICHs must be considered. Sterilization remains a key translational bottleneck. Gamma irradiation has been evaluated in alginate hydrogels [301], whereas UV treatment has been reported for PEDOT hydrogels before cell testing [37]. Steam and ethylene oxide offer alternative routes but require evaluation of network alterations and residual sterilants [301]. Future studies should establish formulation-specific sterilization protocols that ensure microbial inactivation while preserving cytocompatibility, injectability, conductivity, and degradation behavior; laboratory treatment alone does not demonstrate clinical sterility assurance.

Clinical translation also requires traceable feedstocks and batch consistency. Natural matrices include tissue-derived collagen/gelatin, algae-derived alginate, and chitin-derived chitosan [209], whereas PEG and conducting polymers are synthesized from defined monomers. MAX-derived MXene nanosheets and metal-salt-derived Ag nanostructures provide conductive pathways for regenerative scaffolds and bioelectronic interfaces [61,64]. Variations in biological source, extraction, molecular weight, and functionalization can affect matrix properties; filler morphology, oxidation, and dispersion likewise require standardization. Synthetic components can offer greater compositional control, tunability, and experimental reproducibility [302], but are not intrinsically superior in biological performance. Future ICHs should combine synthetic controllability with the bioactivity of selected natural components, supported by validated batch-release criteria. The materials comprising ICHs should be compatible with clinically approved injection devices, maintain stable rheological and electrical properties post-sterilization and storage, and facilitate a straightforward injection process.

Conductive stability and transport characterization under physiological conditions. It is crucial to monitor ICH conductivity in moist environments and under dynamic stretching, enzymatic degradation, swelling, and electrical stimulation. The high conductivity observed under static in vitro conditions may not be maintained within the dynamic environment of muscle tissue. Four-point-probe measurements reduce contact-resistance errors but do not intrinsically separate ionic and electronic conduction or eliminate polarization artifacts. Electronic transport depends on connected conductive domains, whereas ionic transport involves mobile ions in hydrated channels. Under DC bias, ion redistribution and electrode polarization can produce time-dependent apparent resistance. Future studies should combine EIS with physically justified transport models [96] and, where appropriate, DC polarization using validated ion-blocking contacts while excluding Faradaic leakage. Hydration, electrolyte composition, temperature, electrode geometry, applied bias, and measurement duration should be specified; a single conductivity value cannot establish the dominant transport mechanism.

Tissue architecture and functional integration. Furthermore, skeletal muscle has a highly organized architecture, with aligned myofibers, an extracellular matrix, blood vessels, and neuromuscular junctions. Consequently, the development of next-generation ICHs should progress beyond functioning as conductive gels to encompass anisotropic, patterned, or mechano-responsive hydrogels. Techniques such as shear-induced alignment, magnetic or electrical field alignment, injectable microgel assembly, three-dimensional bioprinting, and mechano-electrical conversion may be used to construct hydrogels that replicate the detailed structure and function of skeletal muscle. Recent advances in alginate/carbon-nanotube hydrogels integrate flexible strain sensing with swelling-driven 4D fabrication of nerve guidance conduits [303]. Extending this strategy to VML repair could enable defect-adaptive, anisotropic scaffolds supporting myofiber alignment, reinnervation, and mechanical monitoring, although regenerative efficacy and conductive-network stability require validation in muscle-defect models. Furthermore, by integrating ICHs with external electrical stimulators, wearable bioelectronics, or closed-loop physiotherapy systems, innovative approaches in the personalized repair of skeletal muscle tissue may be developed.

In conclusion, ICHs represent a promising class of multifunctional biomaterials for skeletal muscle repair. Their significance lies in their injectability, conductivity, and integration of material structure, microenvironmental regulation, and functional bioelectrical reconstruction. With continued advancements in conductive component design, dynamic network engineering, biosafety evaluations, and functional assessments, ICHs will become a pivotal therapeutic platform for transitioning skeletal muscle regeneration from structural repair to functional recovery.

## Figures and Tables

**Table 1:** Representative compositions, principal advantages, and key limitations of cross-linking mechanisms relevant to injectable conductive hydrogel design.

Cross-Linking Mechanism	Representative Components	Principal Advantages	Key Limitations	Ref.
Hydrogen bonding	MXene/PEDOT:PSS/tannic acid within a thiol–maleimide PEG network	Reversible interfacial association supports conductive-phase integration and energy dissipation.	Hydration competes with polymer association; sufficient retention can require complementary junctions.	[99,157]
Hydrophobic self-assembly	DBSA-mediated PEDOT:PSS assembly; PEDOT:PSS/Pluronic F127	Dynamic or thermoresponsive assembly supports injection and conductive-domain organization.	Excessive association promotes phase separation; performance depends on temperature and composition.	[38,115,120]
π–π stacking	Aromatic/conjugated PEDOT-rich domains in DBSA-mediated assemblies	Cooperates with hydrophobic association to organize conductive pathways and enable network recovery.	Requires suitable aromatic domains; stacking may promote aggregation without interfacial control.	[38,120]
Electrostatic interactions	PEDOT:PSS with PANI, PPy, or amino-functionalized polyindole	Aqueous charge-driven assembly couples network formation with conductive-polymer integration.	Ionic screening and pH-dependent charge states can alter association and retention.	[99,136]
Host–guest interactions	Adamantane-modified sulfated alginate/PEDOT and poly(β-cyclodextrin)	Specific reversible recognition enables injection, self-healing, and competitively triggered cell recovery.	Requires matched functionalized partners; triggered disassembly does not establish physiological resorption.	[141]
Thiol–Michael addition	Thiol-functionalized PEG with acrylate or maleimide PEG; F-rGO or MXene/PEDOT:PSS	Covalent gelation provides structural retention and immobilization of conductive components.	Biodegradability depends on backbone and cleavable-linker chemistry; covalent gelation alone does not ensure resorption.	[57,157]
Schiff-base reaction	Aminated HA–PANI/oxidized HA; gelatin/oxidized xanthan gum with PDA-reduced graphene oxide	Dynamic imine junctions support aqueous gelation, self-healing, and tissue-adaptive remodeling.	Hydrolytic stability depends on imine chemistry and local conditions; gelation must be coordinated with cell delivery.	[99,146,160]
Boronic acid esterification	PVA/phenylboronic acid-modified oxidized HA with MXene@Mg^2+^	Reversible boronate ester junctions enable network remodeling and stimuli-responsive recovery.	pH and competing diols affect junctions; responsiveness can compromise prolonged retention.	[164,166]
Metal–ligand coordination	Gallol-modified HA microgels with silver-containing interparticle junctions	Reversible interparticle association supports injectability and conductive tissue bridging.	Junction dissociation can reduce conductivity; metal release and particle clearance require evaluation.	[148]
Physicochemical hybrid cross-linking	Thiol–maleimide PEG/MXene/PEDOT:PSS/tannic acid; HCS/TBA/Fe^3+^/PPy	Combines dynamic recovery with structural reinforcement, conductive continuity, and interfacial adhesion.	Multiple interacting components complicate control of gelation, persistence, and biological-effect attribution.	[157,182]

**Table 2:** Reported injectability parameters and electrical characteristics of representative injectable conductive hydrogels.

ICH System	Gelation Time	Injection Conditions	Yield Stress	Other Reported Conductivity (S/m)	Before Injection (S/m)	After Injection/Extrusion (S/m)	Ref.
FPCP	NR (gel at 37 °C)	Injectable; gauge/force NR	NR	0.48 ± 0.03 (formed gel)	NR	NR	[8]
HGC-MX	NR (sol–gel transition ~30 °C)	Injectable; gauge/force NR	NR	0.08 (formed gel)	NR	NR	[184]
RT-PEDOT:PSS	~10 min (4 vol% DBSA; tube inversion)	Syringe-injected precursor; gauge/force NR	NR	~10 (formed gel)	NR	NR	[38]
DECPLMH	NR (bulk recovery ≤5 min after extrusion)	27 G; force NR	NR	NR (bulk electrical tests reported without extractable paired value)	NR	NR	[144]
MeHA-Ga/AgNP granular gel	NR (preformed jammed microgels)	27 G; force NR	Shear yielding observed; value NR	5.0 ± 0.3 (granular cylinders)	NR	~5 (extruded filaments; not paired)	[148]
PEDOT:AlgS/alginate ink	NR (FeCl_3_ treatment 5 min; not gelation time)	27 GA tapered tip; 5 psi printing pressure; force NR	NR	~0.075 (ink, before ionic cross-linking)	NR	NR	[37]
ICAA	42.4–252.1 s (formulation-dependent)	Injectable precursor; gauge/force NR	NR	92.43 ± 7.65 (fully swollen gel)	NR	NR	[157]

Notes. NR, not reported in the verified accessible article text.

**Table 3:** Functional advantages and potential limitations of injectable conductive hydrogels.

Versatility	Principal Advantages	Potential Limitations
Shear thinning	Enables minimally invasive extrusion and conformal filling of irregular VML defects; lowers injection force [221].	Very low viscosity or yield stress causes leakage and poor retention, whereas excessive values cause clogging, high injection pressure, and tissue or cell damage [221].
Self-healing	Restores network integrity and conductive pathways after injection or cyclic muscle deformation [222].	Dynamic bonds may reduce modulus or long-term stability, and recovery can be incomplete or slow under physiological conditions [223].
Tissue adhesion	Improves wet-site retention, closes interfacial gaps, and lowers electrical contact impedance [157].	Excessive adhesion may damage tissue during removal; durability can decline in blood or exudate and during repeated motion [224].
Physiological stability	Maintains geometry, mechanics, conductivity, and interfacial contact in bodily fluids [157].	Dense or anti-swelling networks may restrict nutrient diffusion, cell infiltration, and degradation, or reduce injectability [157].
Biodegradability	Hydrolytic cleavage of labile cross-linkers [57]. and enzymatic cleavage of susceptible polymer backbones enable matrix remodeling and tune scaffold persistence to regeneration [146,212].	Rapid degradation compromises mechanics and conductivity, whereas persistent fillers or degradation products may cause toxicity or inflammation [225].
Biocompatibility	Supports myogenic, vascular, neural, and immune cells; cell or growth-factor loading further improves local retention and provides sustained biological signaling [28].	Safety is formulation- and dose-dependent; gelation or injection shear may impair cell viability, while diffusion limits, burst release, cargo instability, residual cross-linking dopants, and filler fate require evaluation [226].

**Table 4:** Representative injectable conductive hydrogel systems with direct in vivo evidence for skeletal muscle defect regeneration.

ICH System	Network Formation	Conductive Component	Conductivity (mS cm^−1^; Conditions)	Physiological Stability	In Vivo Model and Protocol	Main Bioactive Functions and Regenerative Evidence	Application	Ref.
FPCP	Dynamic Schiff-base crosslinking; F127 thermogelation	PPy@PDA	4.8 ± 0.3; four-point probe (1 mA; 1 mm spacing)	In vivo degradation NR	Female SD rats, 180–200 g; TA full-thickness defect, 5 × 3 × 2 mm^3^ (~10 mg); 50 μL; 1/4 wk; ES NR	Antioxidant/photothermal; ↑MyoD, MyoG, and MHC; H&E/Masson: ↑centronucleated fibers, ↓fibrosis; ↓IL-6; ↑CD31^+^ vessels; innervation/force NR	Full-thickness TA muscle regeneration	[8]
GKP-8	Dynamic Schiff-base bonds, H-bonding, and electrostatic interactions	PDA-functionalized PPy nanoparticles (0.4%)	NR; four-point probe at room temperature (RTS-8.4)	In vivo biodegradation demonstrated; kinetics NR	Female SD rats, 6 wk, 200–220 g; streptozotocin-induced diabetic quadriceps VML, 10 × 6 × 4 mm^3^; 500 μL; 4 wk; no therapeutic ES (1–10 V for assessment only)	M2-like polarization (↑Arg-1/CD206, ↓iNOS/IL-1β); ↑MHC^+^/centronucleated fibers; ↓fibrosis; improved electromyography, gait/climbing, and tetanic force (1.31 N); vessels/innervation NR	Diabetic VML repair via immunomodulation/electrocoupling	[218]
OHA/AHA-PANI_2_/Leu	Dynamic Schiff-base network; self-healing	HA-grafted PANI	0.54–1.18; conditions NR	In vivo biodegradable; kinetics NR	Rats; TA defect; strain/sex, size, dose, and follow-up NR; Leu-loaded hydrogel; ES NR	Cell recruitment/delivery; antioxidant; ↑myogenic differentiation and gene expression; ↓inflammatory factors; vessels/innervation/force NR	TA muscle-defect/VML repair	[160]
GT/rGO_4_ microcryogel + MDSCs	Chemical crosslinking during cryogelation; injectable porous microcryogels	rGO (4 mg mL^−1^)	NR	Biodegradable; kinetics NR	Mice; TA VML; strain/sex, size, dose, and follow-up NR; MDSC-loaded microcryogels; ES NR	Protected injected cells; ↑myoblast growth/differentiation, new myofibers, and vessels; ↓collagen/inflammation; innervation/force NR	MDSC delivery for VML repair	[9]
HM/GM/PFeC/Trp	Photo-crosslinked HAMA/GelMA; magnetic alignment before network fixation	PDA-chelated CNT–Fe_3_O_4_	1.3–1.5; conditions NR	Complete in vitro degradation in PBS at 27–69 d (composition-dependent)	SD rats; TA defect; size, dose, and follow-up NR; HM/GM/PFeC/Trp; ES NR	Anisotropic alignment; Trp-assisted myogenesis; ↑regeneration and myogenic markers; ↓inflammation; vessels/innervation/force NR	Anisotropic TA muscle-defect repair	[50]
AHA-AT_10_%/GT/OPU/IGF-1/VEGF	Schiff-base/acylhydrazone dynamic bonds plus physical IPN; unidirectional freezing and cyclic freeze–thaw	Aniline tetramer (AT)	NR	Biodegradable; sequential IGF-1/VEGF release for ≥12 d	Rats; TA VML; defect size/dose NR; cryogel ± IGF-1/VEGF; 1/4 wk; ES NR	Sequential IGF-1/VEGF delivery; ↑regenerating myofibers and capillaries; ↓inflammation; innervation/force NR	Dual-growth-factor VML repair	[10]
OPTN	Dynamic acylhydrazone and Schiff-base bonds; redox-responsive disulfides	PEI-grafted Nb_2_C MXene@PDA	2.00; conditions NR	Redox-responsive degradation; in vivo kinetics NR	Rats; TA VML; defect size, dose, and follow-up NR; OPTN; ES NR	ROS scavenging; M2-like response; ↓inflammation/fibrosis; ↑myotubes, angiogenesis, and neurogenesis; functional recovery (force metric NR)	Structure–functional VML repair	[28]
FPGM	Dynamic Schiff-base crosslinking; F127 hydrophobic thermogelation	PEI-functionalized Ti_3_C_2_T_x_ MXene	9.7 ± 0.18; conditions NR	Almost completely degraded by day 14 in vivo	Female rats, 180–200 g; TA full-thickness defect, 7 × 3 × 4 mm^3^; 50 μL; 4 wk; ES NR	ROS scavenging; autophagy; M2-like response; ↑myofibers, vessels, and innervation; ↓fibrosis; ↑CMAP/conduction; force NR	Muscle-defect repair with innervation	[66]
L6/MXene–adECM (AM300)	Thermoresponsive physical self-assembly of adECM at 37 °C	Ti_3_C_2_T_x_ MXene (300 μg mL^−1^)	0.456 ± 0.074; four-point probe (converted from 45.63 ± 7.36 mS m^−1^)	>80% mass loss by day 15 in PBS at 37 °C (~50% by days 7–9)	Male SD rats, 8 wk; TA VML, 7 × 10 × 3 mm^3^ (~30% TA); 300 μL + L6 cells (8 × 10^6^ mL^−1^); 7/14/30 d; no therapeutic ES	↑MHC^+^/laminin^+^ fibers and CD31^+^ vessels; ↓collagen; ↑Tuj1^+^ nerves and SV2/α-BTX neuromuscular junctions; improved gait and tetanic force (0.082 ± 0.012 N)	Cell-assisted VML repair with reinnervation	[263]
HGC-MX	Thermoresponsive physical gelation through hydrophobic association (~30 °C)	Ti_3_C_2_T_x_ MXene (3 mg mL^−1^)	0.8; conditions NR	NR	Mice; VML (site/size NR); HGC-MX injection; dose/follow-up NR; external ES (parameters NR)	↑Fiber maturation/muscle strength; ↓fibrosis; ES further ↑angiogenesis; innervation NR	VML repair with ES	[184]

Notes: Only injectable or needle-deliverable conductive hydrogels/cryogels with direct in vivo evidence in traumatic skeletal muscle-defect or VML models were included. Conductivity values were converted to mS cm^−1^ where necessary and are not directly comparable unless measurement conditions are identical. NR, not reported; TA, tibialis anterior; VML, volumetric muscle loss; ES, electrical stimulation; SD, Sprague–Dawley; MDSCs, muscle-derived stem cells; CMAP, compound muscle action potential; adECM, adipose-derived decellularized extracellular matrix; ↑, increased; ↓, decreased. Additional abbreviations: PPy, polypyrrole; PDA, polydopamine; PANI, polyaniline; OHA, oxidized hyaluronic acid; AHA, aminated hyaluronic acid; Leu, leucine; rGO, reduced graphene oxide; HAMA, hyaluronic acid methacrylate; GelMA, gelatin methacryloyl; CNT, carbon nanotube; Trp, tryptophan; AT, aniline tetramer; IPN, interpenetrating polymer network; IGF-1, insulin-like growth factor 1; VEGF, vascular endothelial growth factor; PEI, polyethylenimine; ROS, reactive oxygen species; MHC, myosin heavy chain; iNOS, inducible nitric oxide synthase; Arg-1, arginase-1; Tuj1, class III β-tubulin; SV2, synaptic vesicle protein 2; α-BTX, α-bungarotoxin. Formulation codes (e.g., FPCP, GKP-8, OPTN, FPGM, HGC-MX, PFeC, and OPU) are retained as reported in the source studies.

## Data Availability

No new data were created or analyzed in this study. Data sharing is not applicable to this article.
